# Functional dissection of human mitotic genes using CRISPR–Cas9 tiling screens

**DOI:** 10.1101/gad.349319.121

**Published:** 2022-04-01

**Authors:** Jacob A. Herman, Sonali Arora, Lucas Carter, Jun Zhu, Sue Biggins, Patrick J. Paddison

**Affiliations:** 1 Howard Hughes Medical Institute, Basic Sciences Division, Fred Hutchinson Cancer Research Center, Seattle, Washington 98109, USA;; 2 Human Biology Division, Fred Hutchinson Cancer Research Center, Seattle, Washington 98109, USA;; 3 Department of Genetics and Genomic Sciences, Icahn Institute of Genomics and Multiscale Biology, Icahn School of Medicine at Mount Sinai, New York, New York 10029, USA

**Keywords:** CRISPR–Cas9, functional genomics, human genome, human proteome, mitosis, kinetochore, spindle assembly checkpoint, MAD1L1, Mad1

## Abstract

The identity of human protein-coding genes is well known, yet our in-depth knowledge of their molecular functions and domain architecture remains limited by shortcomings in homology-based predictions and experimental approaches focused on whole-gene depletion. To bridge this knowledge gap, we developed a method that leverages CRISPR–Cas9-induced mutations across protein-coding genes for the a priori identification of functional regions at the sequence level. As a test case, we applied this method to 48 human mitotic genes, revealing hundreds of regions required for cell proliferation, including domains that were experimentally characterized, ones that were predicted based on homology, and novel ones. We validated screen outcomes for 15 regions, including amino acids 387–402 of Mad1, which were previously uncharacterized but contribute to Mad1 kinetochore localization and chromosome segregation fidelity. Altogether, we demonstrate that CRISPR–Cas9-based tiling mutagenesis identifies key functional domains in protein-coding genes de novo, which elucidates separation of function mutants and allows functional annotation across the human proteome.

## Abstract (precis)

In this Resource/Methodology, Herman et al. developed a method that leverages CRISPR–Cas9-induced mutations across protein-coding genes for the a priori identification of functional regions at the sequence level. As a test case, they applied this method to 48 human mitotic genes, revealing hundreds of regions required for cell proliferation, including domains that were experimentally characterized, ones that were predicted based on homology, and novel ones.

The sequencing and characterization of the human genome (Lander et al. 2001; The ENCODE Project Consortium 2012) has provided a reliable list of >20,000 protein-coding genes (e.g., UniProtKB database) (Breuza et al. 2016). However, our current understanding of how protein activities are compartmentalized into distinct functional domains mostly relies on homology-based comparative genomics. For example, the human proteome contains 5494 separate conserved protein family (Pfam) domains, each with a putative function (e.g., the methyltransferase-like domain) (Mistry et al. 2013); however, >3000 of these domains have unknown function (Bateman et al. 2010), and about half of the proteome is entirely unannotated (Punta et al. 2012; Mistry et al. 2013; El-Gebali et al. 2019). Moreover, many protein-coding genes are inscrutable to homology-based annotation methods because disordered protein regions are only conserved among the most similar of species yet perform critical cellular functions (Gsponer and Babu 2009; van der Lee et al. 2014; Ota and Fukuchi 2017). To this point, within the human genome, long disordered regions are the least likely sequences to be recognized as a Pfam domain (Mistry et al. 2013). Without methods to resolve the subfunctionalization of human proteins independent of homology-based inference, we lack the ability to fully characterize these genes.

Current gene manipulation technologies, such as RNAi (Paddison and Hannon 2002; Paddison 2008) and CRISPR–Cas9 (Mali et al. 2013; Shalem et al. 2014), although powerful, do not readily resolve the multifunctional nature of protein-coding genes. Instead, in their most common forms, these technologies attenuate total gene activity via knockdown, knockout, or transcriptional repression and fail to provide insight into a protein's domain architecture or features. However, this important gap in knowledge may be addressed using an infrequently appreciated CRISPR–Cas9-based approach: tiling sgRNA mutagenesis. This approach was initially used to define new design rules for sgRNAs (Shi et al. 2015; Munoz et al. 2016). These pooled sgRNA outgrowth screens, where many sgRNAs targeted each protein-coding gene, revealed that the sgRNAs causing the most significant changes in outgrowth targeted Pfam domains (Munoz et al. 2016). While these approaches suggested that tiling mutagenesis reveals the functional landscape of protein-coding genes, they did not extend this analysis to identify novel critical regions within the coding DNA sequences (CDS). Moreover, approaches that leverage dCas9 to recruit mutagenizing enzymes like promiscuous deaminases tend to mutagenize only 5%–15% of alleles, making them useful for identifying gain-of-function mutations but less applicable for loss-of-function mutations where the high proportion of wild-type alleles obscures phenotypes (Hess et al. 2016; Ma et al. 2016).

Tiling mutagenesis works because, in somatic cells, Cas9:sgRNA complexes induce dsDNA breaks that are commonly repaired by error-prone nonhomologous end joining (NHEJ), leaving repair scars in the form of small insertion/deletion (indel) mutations (Lieber et al. 2003; Hartlerode and Scully 2009). Recent deep sequencing of >100 protein-coding loci in human cells targeted by Cas9 indicates that, on average, 80% of mutations occur with 1n or 2n nucleotides inserted or deleted and thus shift the triplet reading frame (Chakrabarti et al. 2019). Therefore, when a single sgRNA targets a population of diploid cells, 64% of cells should harbor biallelic frameshift mutations, while the remaining 36% of cells will carry at least one in-frame but mutagenized allele (Fig. 1A; Supplemental Fig. S1A).

**Figure 1.**
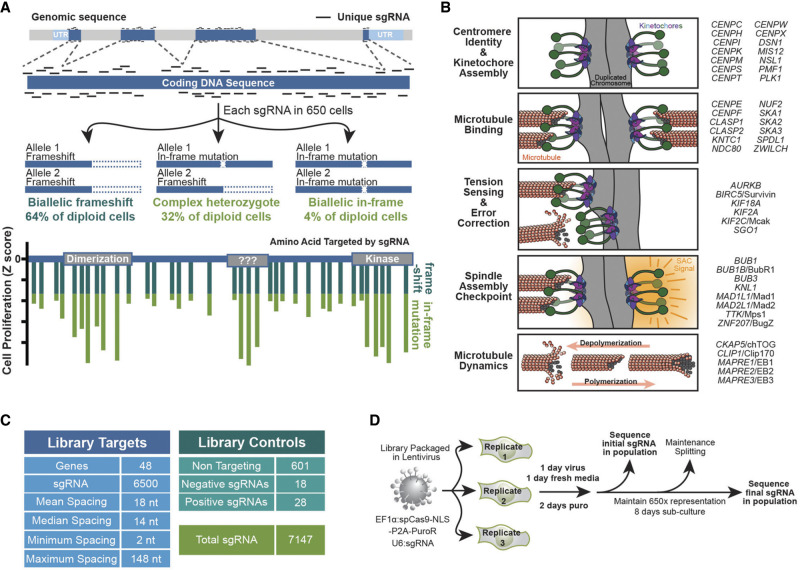
Design and execution of a CRISPR/Cas9 tiling screen. (*A*) Schematic of a CRISPR–Cas9 tiling screen and phenotypic readout. The percent of population containing each genotype is calculated based on in-frame repairs occurring at 20% of edits (Chakrabarti et al. 2019), and a hypothetical example of how this reveals functional protein motifs is shown *below*. (*B*) Cartoon representation of key molecular activities of kinetochore- and microtubule-mediated processes. Genes/proteins to be screened are listed at the *right* according to their best-characterized function. (*C*) Characteristics of a tiling library targeting mitotic factors. (*D*) Schematic of a proliferation-based screening approach using lentiviral particles to insert both spCas9 and sgRNA sequences into genomic DNA. This allows next-generation sequencing of sgRNA sequences to serve as an indirect readout of their effect on cell growth.

This mutagenic behavior reveals critical protein domains because their residues are phenotypically constrained and less mutable than other genic regions. This means sgRNAs targeting constrained gene regions in an outgrowth screen will affect the most dramatic dropout and will be recognized as “peaks” when next-generation sequence analysis is displayed along the CDS as hypothetically displayed in Figure 1A. Historically, mutagenesis strategies in mammalian cells have relied on ectopic overexpression of mutant proteins with unclear physiological relevance, while tiling mutagenesis targets the genomic locus and thus maintains physiological protein regulation.

To rigorously test the ability of CRISPR–Cas9 tiling libraries paired with outgrowth screens to elucidate functional protein sequences, we required a set of well-studied, highly multifunctional factors. Such a set of proteins would already be annotated for many critical, experimentally verified motifs and would enable rapid biological characterization of previously unknown functional regions revealed by tiling. Thus, we targeted factors that ensure mitotic chromosome segregation by regulating kinetochores and microtubules. Kinetochores are large multisubunit complexes that assemble on centromeres and link chromosomes to the dynamic microtubules of the mitotic spindle. During mitosis, the kinetochore–microtubule attachment physically powers chromosome movements and regulates the spindle assembly checkpoint (SAC), which is a biochemical surveillance mechanism that prevents chromosome segregation errors (Fig. 1B; London and Biggins 2014b; Musacchio 2015; Hara and Fukagawa 2020). Decades of study have revealed much of the underlying chemical and physical properties that enable kinetochore assembly, attachment to microtubules, and SAC surveillance, yet we still do not fully understand the multifunctional nature of these factors.

Here, we selected 48 mitotic genes to target (Fig. 1B). A comprehensive literature review revealed these proteins contained 167 experimentally defined functional regions and 96 Pfam domains, yet >50% of the coding sequence fell outside of these areas, indicating a chance to reveal novel domains. By performing sgRNA tiling screens in multiple cell lines, we identified hundreds of putative essential regions among these genes. Approximately 65% of these regions overlap with literature-defined functional regions or Pfam domains, while the remaining approximately one-third of functional regions identified by tiling have not been studied. Consistent with technological limitations associated with interrogating disordered and evolutionarily divergent sequences, the “novel” functional regions have significant overlap with these rarely interrogated domains. We validated 15 of these functional regions appearing across six genes and further characterized the biological role of a previously unknown domain in the SAC protein *MAD1L1*/Mad1.

## Results

### Generation of a CRISPR–Cas9 tiling library targeting mitotic factors

We designed an sgRNA tiling library in silico that targeted 48 mitotic factors spanning biological functions and genomic contexts (Fig. 1B), including two paralogous gene sets: *CLASP1/2* and *MAPRE1/2/3* (Komarova et al. 2005; Mimori-Kiyosue et al. 2005; Pereira et al. 2006). With genes chosen, we then identified all the unique sgRNA targeting each CDS that (with a few exceptions) did not target other coding regions of the genome (Supplemental Fig. S1B). This resulted in a library of 6500 sgRNAs with median spacing of 14 nt between cut sites within the CDS and a maximum spacing of 148 nt due to a lack of the spCas9 protospacer-adjacent motif (PAM; NGG) (Fig. 1C).

Using two different predictors for repair bias after CRISPR–Cas9 editing, we found that the library, on average, did not contain any positional bias for sgRNAs predicted to favor frameshifting edits (Supplemental Fig. S1C,D; Shen et al. 2018; Chakrabarti et al. 2019). However, within a single gene some bias could be observed, particularly within short genes (<300 amino acids) that were targeted with only 30–40 sgRNA, suggesting the library may underreport in these instances (Supplemental Fig. S1E,F).

We also included 601 nontargeting control (NTC) sgRNA sequences that cause no editing in the human genome. Thus, NTC sgRNAs reported the rate of unperturbed proliferation against which mitotic-specific sgRNAs were compared (Sanjana et al. 2014). Finally, to monitor screen performance, we also included a small collection of sgRNAs targeting genes that were previously shown to positively (*CDKN2A*, *TP53*, etc.) or negatively (*POLR2L*, *HEATR1*, etc.) regulate proliferation (Toledo et al. 2015; O'Connor et al. 2021). This final library contained 7147 sgRNAs (Fig. 1C) that were synthesized as a pool and inserted into an “all-in-one” single lentiviral expression vector.

We infected three independent replicates of cells such that spCas9 and each sgRNA were incorporated into the genome of 650 cells (Fig. 1D). The sgRNA sequences were PCR-amplified from genomic DNA of populations harvested immediately after infection and after 8 d of outgrowth. Each sgRNA was identified by Illumina sequencing to determine how its representation altered over the 8 d of proliferation. The change in normalized sequencing reads for each sgRNA was used to calculate a median log_2_(fold change) for each cell line and convert that to a *Z*-score (Supplemental Table S1).

### The tiling proliferation screen is reproducible, and most potent when targeting functional protein regions

**Figure 2.**
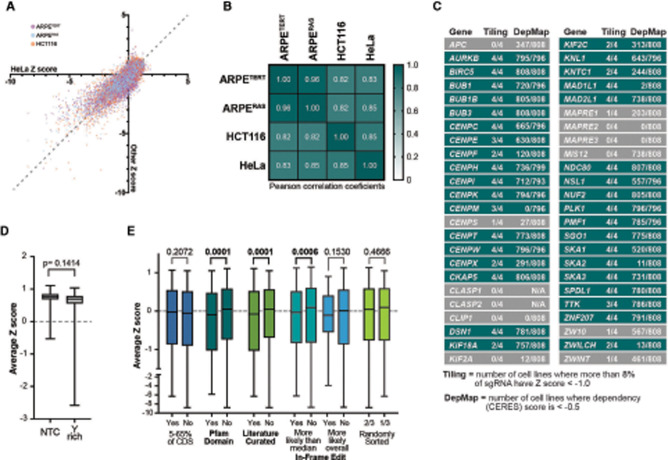
The CRISPR/Cas9 tiling screen is technically and biologically reproducible, and sgRNA dropout is associated with targeting functional protein domains. (*A*) Each sgRNA's *Z*-score from three cell lines—ARPE^TERT^, ARPE^RAS^, and HCT116 cells—is plotted relative to the *Z*-score in HeLa cells. These data exclude nontargeting controls, and the dashed line (*y* = *x*) is plotted for reference. (*B*) Correlation matrix and heat map for all targeting sgRNA in each cell line with Pearson correlation coefficients displayed. (*C*) Table of 48 genes tiled in the screen. The “Tiling” column reports the number of cell lines in which >9% of sgRNA targeting that gene had a *Z*-score less than −1.0, indicating that the gene overall was important for cell proliferation. The DepMap column reports the number of cell lines screened where a gene was given a “dependency score” less than −0.5 at the time of writing, corresponding to a strong negative effect on proliferation. (*D*) Average *Z*-score for nontargeting controls (NTCs) in all four cell lines and targeting sgRNAs that contain the recently reported pyrimidine-rich (Y-rich) sequence at their 3′ ends (Graf et al. 2019). (*E*) Targeting sgRNA, minus those containing the Y-rich sequence, were binned based on which genomic or protein features they targeted. “In-frame edits” were predicted using Indelphi (Shen et al. 2018) and binned into groups above and below the median likelihood of in-frame edits (22.4%) or above and below the overall likelihood of in-frame edits (50%). All box plots show median, quartiles, and range of the average *Z*-score for each sgRNAs among four cell lines. Mann–Whitney tests were used to determine *P*-values.

To ensure that this approach was not driven by unique cellular or genomic contexts (copy number variations, doubling rates, etc.), we performed the tiling screen in four diverse cell types. This included common cell lines HeLa (aneuploid) and HCT116 (near diploid), as well as a *TERT* immortalized retinal pigment epithelial cell line (ARPE^TERT^; diploid) and a laboratory transformed derivative with numerous genetic alterations, including an ectopic copy of oncogenic *HRAS* (ARPE^RAS^; aneuploid). Despite these unique cellular backgrounds, on-target sgRNAs (nontargeting controls excluded) affected proliferation similarly in all cell types (Fig. 2A,B). The sgRNAs affected proliferation in ARPE^TERT^ and ARPE^RAS^ cells extremely similarly (Pearson coefficient 0.96), as expected from their shared lineage, and even behaved similarly in cells from diverse tissue and disease types (Pearson coefficients >0.81) (Fig. 2A,B). These correlations were also observed when only sgRNAs with the most potent decreases in proliferation were analyzed (bottom quartile) (Supplemental Fig. S2A,B). These results indicated that our techniques were reproducible (data span unique lentiviral preparations and sequencing runs) but, more importantly, that our tiling library had similar phenotypic outcomes among diverse cells lines despite the semirandom nature of DNA damage repair following CRISPR–Cas9 targeting.

The goal of the tiling library was to identify motifs that contribute to the essential activity of mitotic factors, but this first required that we determine which of our targets had a negative effect on cell proliferation at the gene level. We identified all sgRNAs with a *Z*-score less than −1, indicating that these sgRNAs were depleted from the population by at least one standard deviation. Within each gene, 0%–53% of sgRNAs met this threshold (Supplemental Fig. S2C). For downstream analysis, we excluded the 15% of genes with the least effect on proliferation (<8% of sgRNAs had *Z*-score less than −1) (Fig. 2C). Our threshold was also consistent with biological observations; for example, targeting *CLASP1* or *CLASP2* did not affect proliferation because these paralogs function redundantly for most known activities (Mimori-Kiyosue et al. 2005; Pereira et al. 2006). Genes in which more than two cell lines met this threshold are colored teal in Figure 2C and reflected trends observed in DepMap studies, which at this time had performed CRISPR screens with four to six sgRNAs targeting a single gene in >750 cell lines (Meyers et al. 2017; Tsherniak et al. 2017).

However, there were surprising findings, particularly how *MAD1L1*, *MIS12*, *SKA2*, and *CENPM* behaved differently between our screen and DepMap (Fig. 2C). We found that sgRNAs targeting the *MIS12* gene on average failed to meet our gene-level threshold, yet DepMap results showed decreased proliferation in 90% of cell lines after *MIS12* targeting (Fig. 2C), and RNAi inhibition of *MIS12* is known to induce lethal chromosome segregation defects (Goshima et al. 2003). Moreover, consistent with the DepMap study, the other three genes in the Mis12 complex (*DSN1*, *PMF1*, and *NSL1*) all met our threshold. Looking at the distribution of every sgRNA in our library that targeted *MIS12* (Supplemental Fig. S2D), we saw that only four sequences were strongly depleted, and three of those were used in the DepMap library (Sanson et al. 2018). Thus, the DepMap library contains primarily the most penetrant sgRNAs and identified *MIS12* as essential, while in our tiling library the signal from these four sequences is diluted by the ∼80% of MIS12 targeting sgRNAs that had no effect on proliferation. We hypothesize that these sgRNAs failed to cause editing or exhibited repair bias toward the wild-type sequence, and thus the gene overall did not meet our threshold.

We observed the reverse behavior in *MAD1L1*. All four cell lines had negative proliferation outcomes when this gene was targeted, yet DepMap screening suggests that <1% of cell lines are affected (Fig. 2C). We identified four of the six DepMap sgRNA sequences in our data and found that most of those sequences did not affect proliferation, yet with our increased number of sgRNAs we saw that many other sequences had a strong negative effect (Supplemental Fig. S2C; Sanson et al. 2018). We also found that targeting *CENPM* and *SKA2* had negative proliferation outcomes, whereas DepMap data suggest no growth defects (Fig. 2C). This is because DepMap sgRNA sequences for these genes target rare exons (Supplemental Fig. S2E). Thus, the high-density data derived from tiling libraries complement results from other genome-wide approaches and allow interrogation of rare exons and multiple transcripts without confounding the application of the screen at the gene-wide level. Altogether, CRISPR tiling is highly reproducible, results in high-confidence gene-level data, and is rarely limited by biases in CRISPR technology (e.g., inferior performance of *MIS12* sgRNAs).

Having identified 36 genes that were required for wild-type levels of proliferation, we set out to determine whether any global characteristics drove the performance of the sgRNAs targeting these genes—primarily whether targeting functional protein motifs had the most negative effect on proliferation. After synthesizing our library, it was shown that targeting sequences enriched for pyrimidines near the 3′ end of our sgRNA scaffold cause premature polymerase termination (Graf et al. 2019). We identified sgRNAs with these pyrimidine-rich (Y-rich) sequences within our data and confirmed those findings (Fig. 2D). We excluded these sequences from our global analysis and asked which protein or genomic features were associated with sgRNA activity in our outgrowth screen.

First, we tested whether targeting early exons (within 5%–65% of the CDS) resulted in more penetrant loss of activity due to more robust nonsense-mediated decay (Doench et al. 2016; Sanson et al. 2018). We found no association between targeting early exons and sgRNA performance (Fig. 2E). Instead, our data show that sgRNAs targeting functional protein motifs result in the most potent phenotypes, consistent with findings that in-frame edits are not tolerated in essential domains (Shi et al. 2015; Munoz et al. 2016; Michlits et al. 2020). We found that sgRNAs targeting Pfam domains or functional regions annotated directly from literature had, on average, the most negative effect on proliferation (Fig. 2E). Moreover, we found that sgRNAs that are predicted to be more likely overall (>50% of cases) or more likely than the median (>22.4% of cases) to create in-frame edits were associated with decreased proliferation (Fig. 2E). This strongly suggests that proliferation phenotypes in our screen are not driven by frameshift mutations. Together this global analysis is consistent with the observation that in-frame edits caused by repair after CRISPR–Cas9 nuclease activity are common and have the most potent effect on cell proliferation when they occur in an essential region of the CDS.

### Multiple approaches for integrating tiling data within sequence space reveal functional regions

**Figure 3.**
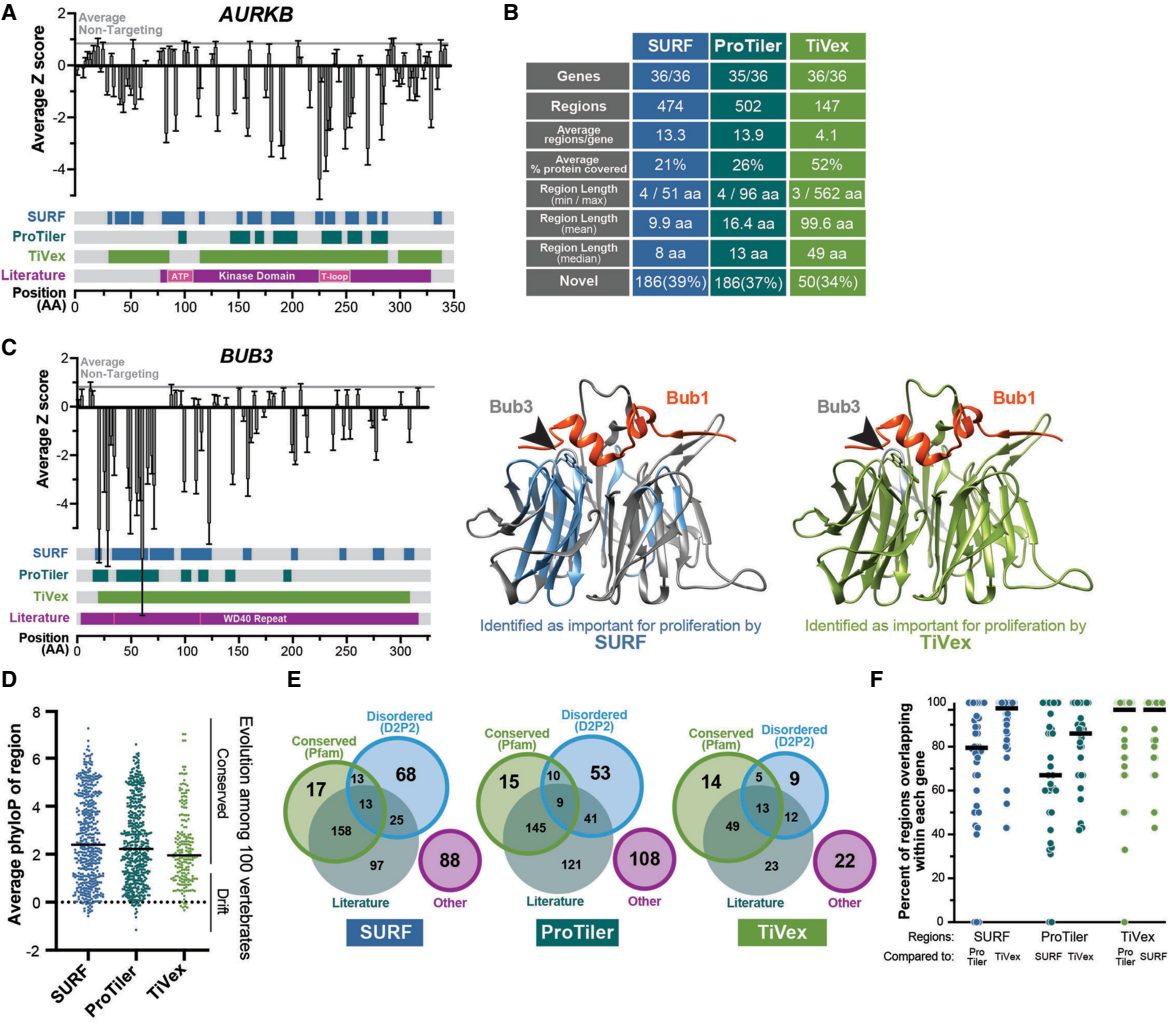
CRISPR/Cas9 tiling data identify previously known and uncharacterized functional regions, including evolutionarily divergent and disordered ones. (*A*) Median *Z*-score from four cell lines for each sgRNA is mapped to where it targets the CDS of *AURKB* (gray vertical bars with 95% confidence interval). Amino acid regions identified by SURF, ProTiler, or TiVex as important for cell proliferation are shown as colored in each track corresponding to coordinates at the *bottom*. *AURKB* functional regions identified in literature are shown in a similar manner; the small pink region in the kinase domain highlights the nucleotide binding pocket and activation loop. The gray horizonal line shows the average value of all nontargeting controls, representing normal levels of proliferation. (*B*) Table describing the characteristics of essential regions identified by analyzing tiling data with SURF, ProTiler, or TiVex in the 36 target genes previously identified (Fig. 2C). (*C*) Sequence-level tiling profile of human Bub3 as in *A* and crystal structures of budding yeast Bub3 bound to a peptide of yeast Bub1 (PDB: 2I3S) are colored based on the homologous regions identified by TiVex (green) and SURF (blue) as important for proliferation (Larsen et al. 2007). The arrowheads highlight two tryptophan residues absolutely required for the protein–protein interaction. (*D*) PhyloP values (based on the alignment of 100 vertebrate orthologs) for each nucleotide within SURF, ProTiler, or TiVex regions were averaged. Bars represent the median, each dot is a region, and “conserved” corresponds to a value of *P* < 0.05. (*E*) Regions identified by SURF, ProTiler, and TiVex as required for proliferation are grouped by their overlap with conserved Pfam domains (Mistry et al. 2013; El-Gebali et al. 2019), functional regions identified in the literature, or predicted disordered domains (Oates et al. 2013). Regions not fitting these categories are grouped as “other” and represent most uncharacterized regions. (*F*) Pairwise analysis of overlap between regions identified by each method. The graph displays the number of SURF regions within a single gene (*left* two columns, blue) that overlap by at least one amino acid with ProTiler or TiVex.

The power of the tiling library is to gain an unbiased understanding of protein function within sequence space. Thus, for each gene, we can display the average *Z*-score for all of the targeting sgRNAs from the four cell lines (Fig. 3A, vertical gray bars) along the translated CDS (Fig. 3A). In *AURKB*, we observed sgRNAs with a strong negative effect on proliferation and sgRNAs that appear largely inactive, since they behave similarly to the average nontargeting controls (Fig. 3A). To integrate these data over the CDS, we used previously published approaches—CRISPR–SURF and ProTiler (Hsu et al. 2018; He et al. 2019)—while also pairing tiling data with a convex fused lasso (TiVex) to generate a more smoothed stepwise function (Parekh and Selesnick 2015). While each method is unique, they all transform tiling CRISPR screen data into a stepwise function and then report ranges of nucleotides or amino acids that are negatively enriched compared with either nontargeting controls or a globally or locally defined “zero” (Fig. 3A, colored regions within each gray bar; Supplemental Table S2). Comparing these methods, we found that TiVex identifies broader boundaries (50–100 amino acids) that better represent discretely folded domains such as a majority of the kinase domain in *AURKB* (Fig. 3A). ProTiler and SURF instead identify multiple, sharper boundaries (10–15 amino acids) within larger domains that may better guide discovery of key functional motifs such as the nucleotide binding pocket or activation loop of *AURKB* (Fig. 3A, pink boxes within *AURKB* kinase domain). In the case of *AURKB*, both SURF and TiVex additionally suggest that an uncharacterized motif within the N terminus also contributes to Aurora B activity. This same trend is observed in larger proteins with multiple folded domains such as *KIF18A*, which is known to encode both a motor domain and separate microtubule binding motif (Supplemental Fig. S3A,B).

Thus, TiVex identified larger windows that overlapped multiple SURF or ProTiler regions and often represented discretely folded protein domains such as the *AURKB* kinase domain, *KIF18A* motor domain, or *CKAP5*/chTOG TOG domains (Fig. 3A; Supplemental Fig. S3A; Supplemental Table S2). This trend was evident among all 36 target genes (Fig. 2B; Supplemental Table S2). SURF and ProTiler identified ∼500 small (10- to 15-amino-acid) regions, and TiVex identified ∼150 large (50- to 100-amino-acid) regions (Fig. 3B). The application of each approach is also demonstrated in three dimensions by mapping SURF or TiVex regions onto the crystal structure of the budding yeast homolog of the target protein Bub3 bound to a fragment of Bub1 (Fig. 3C; Pettersen et al. 2004; Larsen et al. 2007). TiVex identified nearly the entire Bub3 protein as essential because it folds into a single globular structure that contributes to the interaction with Bub1 (Fig. 3C, right green residues). However, SURF primarily identified the two β strands that contain a pair of tryptophan residues that are specifically required for Bub1 binding (Fig. 3C, middle, blue residues) despite these residues being distant in sequence space (Fig. 3C, left small pink boxes in WD40 repeat) (Pettersen et al. 2004; Larsen et al. 2007). Similarly, by mapping TiVex and SURF regions onto a partial structure of the budding yeast homolog of *CKAP5*/chTOG bound to a tubulin dimer (Supplemental Fig. S3B,C), we found that TiVex draws boundaries around the entire TOG domain, while SURF regions instead cluster near the tubulin binding surface.

For some small proteins like Bub3, TiVex identified most of the sequence as important, which is consistent with how the protein functions. We found that, on average, TiVex identifies ∼55% of the protein sequence in each gene as contributing to proliferation (Supplemental Fig. S3D). The same analysis found that Pfam domains or literature-defined functional motifs similarly cover 50%–60% of protein sequences, while SURF and ProTiler methods instead cover 20%–30% of protein sequences (Supplemental Fig. S3D). Thus, TiVex is better suited for characterizing domain boundaries in large proteins that may contain multiple discretely folded functional units, while SURF and ProTiler highlight more precise protein regions that guide further biological exploration and the development of separation of function mutants.

TiVex identified protein domains of a size similar to Pfam yet, unlike Pfam, TiVex was not restricted to conserved protein sequences. We calculated an average conservation score for each region identified by SURF, ProTiler, or TiVex based on the nucleotide conservation among 100 vertebrates (PhyloP) within the UCSC genome browser (Fig. 3D; Kent et al. 2002; Pollard et al. 2010). Approximately 70% of protein motifs identified in the three analysis methods demonstrated some sequence conservation among vertebrate species (*P* < 0.05), while the sequence was not constrained in the remaining ∼30% of protein motifs. The distribution of conservation scores within putative essential regions was also indistinguishable from likely nonessential regions (Supplemental Fig. S3E), further suggesting that CRISPR tiling screens are not limited by evolutionary conservation. When we cross-referenced regions identified by SURF, ProTiler, and TiVex with our manually curated list of functional regions identified in literature, we found that 34%–39% of regions identified by tiling have, to our knowledge, not yet been characterized (Fig. 3B). Some of these unstudied motifs overlapped with conserved regions (Pfam domains), but many of them fell in regions predicted to be disordered or not within either of those categories (Fig. 3E; Supplemental Table S3). Overall, we saw strong agreement between all three analysis methods. In pairwise comparisons, 100% of the protein regions identified by each method overlap for seven to 13 of the genes (Fig. 3F), and major discrepancies are primarily focused in three to four genes like *KNTC1* and *CENPF* (Supplemental Fig. S3F). These differences likely arise from how each method defines “zero” (relative to NTC or gene averages).

As a measure of robustness and to test whether sgRNAs with low editing efficiency could obscure important functional motifs, we performed SURF and TiVex analysis on screen data modified to contain low-efficiency sgRNA. To this end, we generated new data sets by randomly transforming the *Z*-scores for 10%, 20%, 30%, 40%, and 50% of sgRNAs targeting each gene to a value within the range of NTC sgRNAs (Supplemental Fig. S4A). This revealed, in general, that for SURF regions, precision and recall values scale with the amount of nonfunctional sgRNA substitutions but remain robust at 10% data replacement (Supplemental Fig. S4B,C). For TiVex domains, substitution of nonfunctional sgRNAs was robust to 20% replacement, likely owing to the larger size of these regions (Supplemental Fig. S4D,E). However, in either method, many domains were identified even after 50% of the signal was lost. Thus, in tiling outgrowth screens, functional regions may be obscured by low-efficiency sgRNAs but, overall, this outcome is unlikely.

With our new data set, we further confirmed that sgRNA dropout is correlated with targeting functional protein regions (Shi et al. 2015; Munoz et al. 2016), and the most potent sgRNAs are not predicted to favor frameshifting mutations or target an early exon. Instead, we revealed that sgRNAs most strongly affecting proliferation were concentrated within previously characterized functional protein domains and 50–100 putative functional regions of unknown activity.

### Biological validations indicated that CRISPR tiling is highly accurate

**Figure 4.**
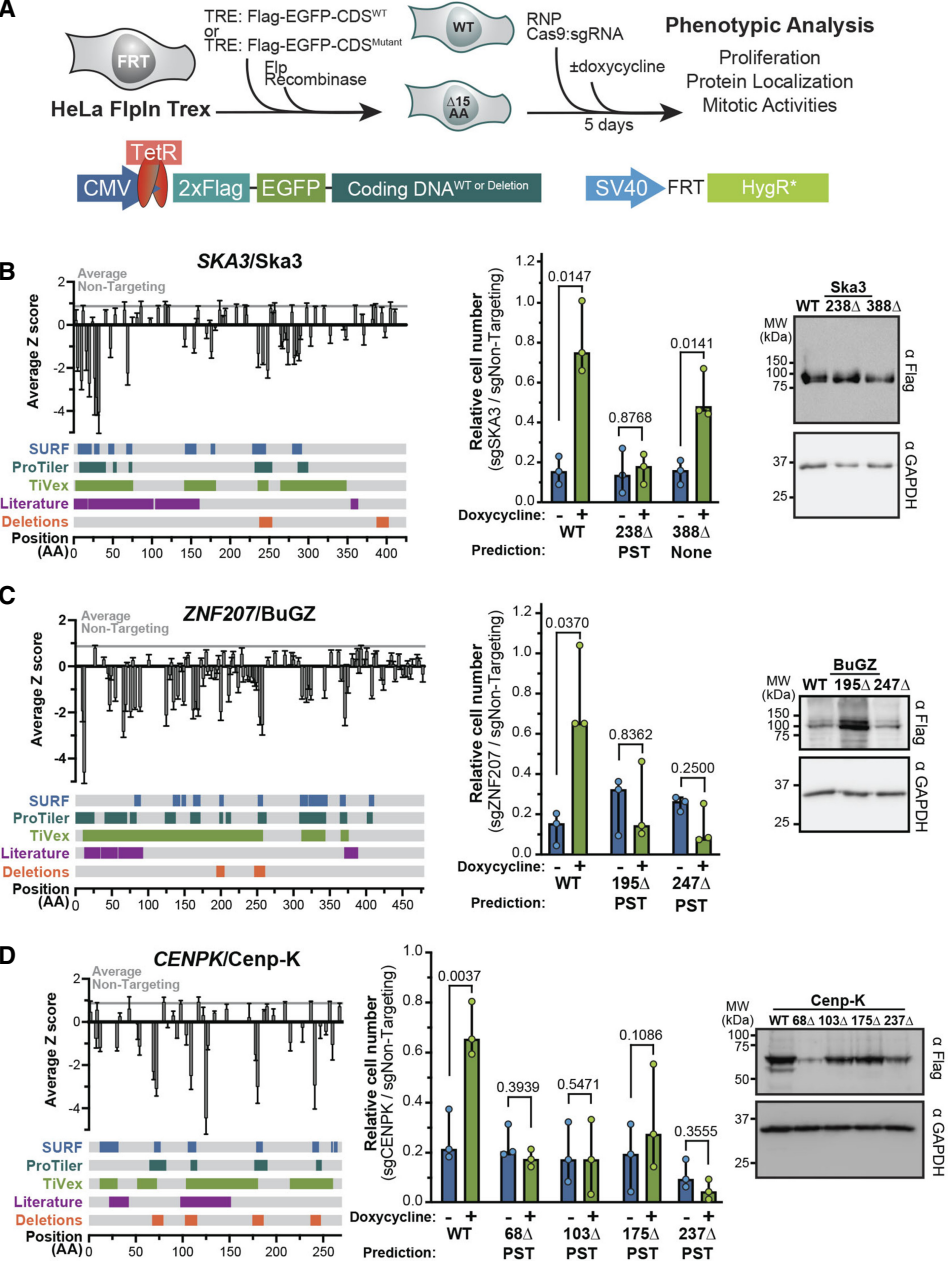
Functional validation and characterization of 11 high-resolution regions within five genes identified by tiling. (*A*) Schematic of generating cell lines in which expression of wild-type or mutant proteins is induced by doxycycline and the CRISPR/Cas9-based complementation approach used for functional validation. (*B–D*) Tiling profile, validation of the proliferation phenotype, and assay of protein stability for *SKA3*/Ska3 (*B*), *ZNF207*/BuGZ (*C*), and *CENPK*/Cenp-K (*D*). Tiling profiles are the same as in Figure 3 while also showing regions that were deleted. Cell proliferation was assayed as the cell number after knockout of endogenous protein relative to a nontargeting control in the presence (green) or absence (blue) of doxycycline. Cell numbers were normalized to the same cell line electroporated with a nontargeting control. The analysis methods predicting a region to be essential shown as P (ProTiler), S (CRISPR–SURF), and T (TiVex). Each dot is a biological replicate, with bars showing median values and 95% confidence intervals. Paired *t*-tests were used to determine *P*-values. Steady-state protein levels of wild-type and mutant proteins were assayed by immunoblot in the presence of endogenous protein, using GAPDH as a loading control and exposed for a shorter interval. For more examples, see Supplemental Figure S4.

Because sgRNA depletion was associated with literature-defined functional motifs, we validated a set of uncharacterized functional regions identified by tiling. We selected 15 uncharacterized regions identified among six genes (*CENPH*/Cenp-H, *CENPK*/Cenp-K, *MAD1L1*/Mad1, *SGO1*/Sgo1, *SKA3*/Ska3, and *ZNF207*/BuGZ), including both highly conserved and evolutionarily unconstrained protein regions (Supplemental Fig. S5A). To test these domains, we generated wild-type proteins with N-terminal 2xFlag and/or EGFP tags and then created small (10- to 40-amino-acid) deletion mutants corresponding to regions identified by SURF, ProTiler, and/or TiVex. Mutant proteins were named for the first residue within the small deletion (Ska3^Δ238–253^, shortened to Ska3^238Δ^ or 238Δ). Transcription of exogenous genes was driven by a highly active doxycycline-inducible promoter (TRE) that was inserted at a unique genomic locus within a parental cell line using a recombinase system (O'Gorman et al. 1991; Gossen and Bujard 1992; Taylor et al. 1998). Cell lines encoding the wild-type or mutant proteins were then electroporated with Cas9 in complex with one to two synthetic sgRNAs that targeted endogenous but not ectopic genes of interest or a nontargeting sgRNA (Fig. 4A; Hoellerbauer et al. 2020a,b). Doxycycline was either withheld or added after electroporation to test the effect of endogenous gene knockout or whether expression of the wild-type or mutant protein complemented its essential activity, respectively.

We tested regions within Ska3, BuGZ, and Cenp-K that were identified by all three computational methods, and one additional region in Ska3 that was not identified in the screen as a control (Fig. 4B–D). In all cases, wild-type proteins provided a significant rescue for cell proliferation following endogenous protein knockout, as did the control deletion in Ska3 (Fig. 4B–D). We observed the same behavior in Cenp-H and Sgo1 deletion mutants that were predicted by all three methods, but also found that a region identified solely by ProTiler was a false positive and was not required for proliferation in our validation study (Supplemental Fig. S5B,C). Altogether, using this complementation approach, we verified that 10 out of 11 ProTiler regions, and 10 out of 10 regions overlapping with SURF and TiVex windows were required for cell proliferation. This comprehensive analysis suggests that CRISPR–Cas9 tiling libraries reliably identify uncharacterized functional protein regions.

### Tiling *MAD1L1*/Mad1 reveals a motif that contributes to its kinetochore localization

**Figure 5.**
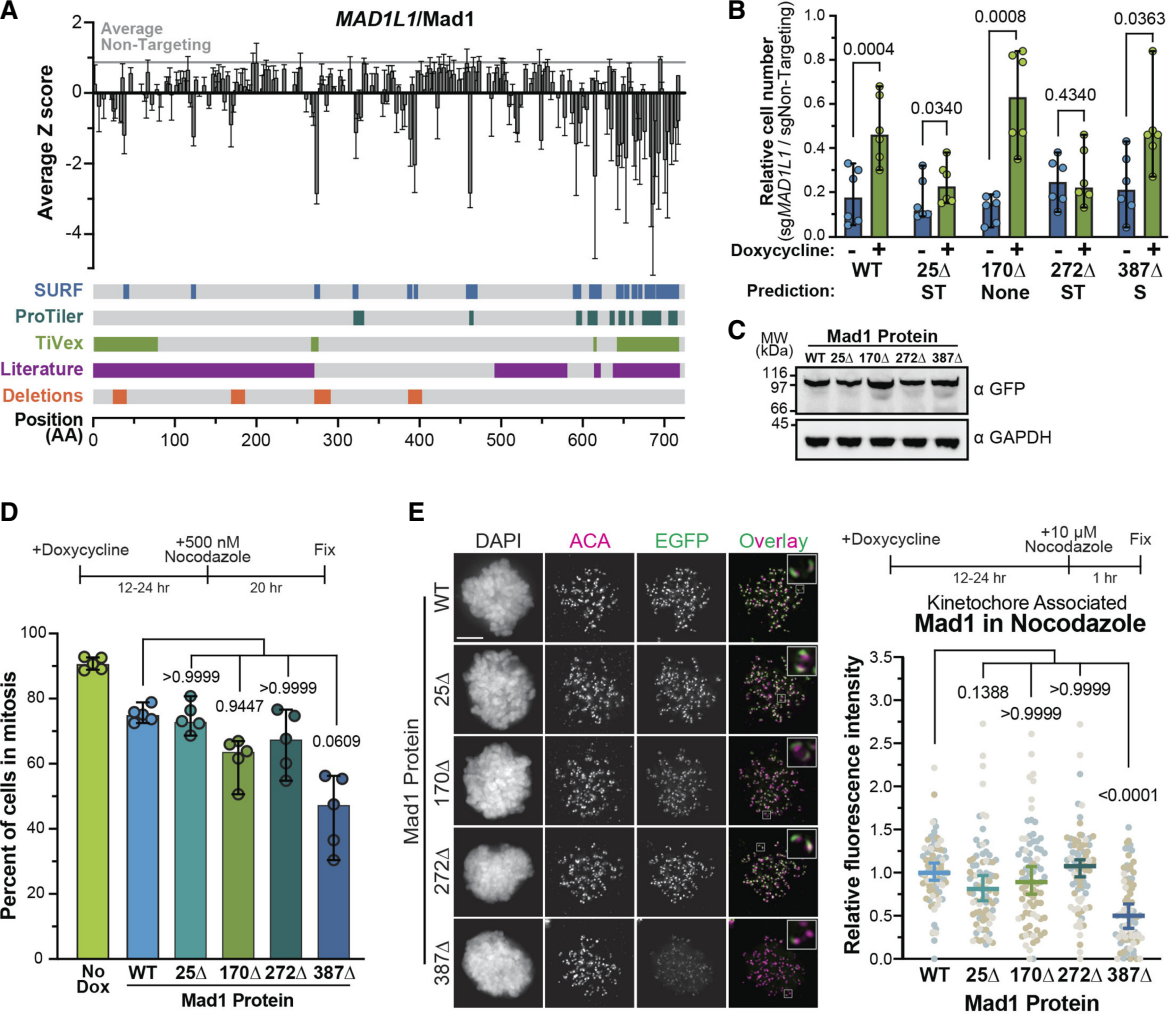
Tiling *MAD1L1*/Mad1 reveals a region contributing to prolonged activation of the SAC and kinetochore localization. (*A*) Tiling profile for *MAD1L1*/Mad1, displayed the same as in Figure 4. (*B*) Normalized cell number after knockout of endogenous protein in the presence (green) or absence (blue) of doxycycline in each cell line. Dots represent three biological replicates performed in duplicate, with paired *t*-tests used to determine *P*-values. (*C*) Steady-state protein levels of wild-type and mutant proteins were assayed by immunoblot in the presence of endogenous protein, using GAPDH as a loading control and exposed for a shorter interval. (*D*) The ability of wild-type or mutant proteins to maintain a prolonged SAC arrest in the presence of endogenous Mad1 was assayed by treating cells with nocodazole for 20 h and determining the percent of cells in mitosis based on chromosome morphology. Data in “No Dox” were determined from all five cell lines not exposed to doxycycline. Each dot represents a biological replicate, and Dunn's multiple comparisons test was used to determine *P*-values. (*E*) Kinetochore association of EGFP-Mad1 wild-type and mutant fusion proteins was determined by the EGFP signal proximal to the anticentromere antibody (ACA) in the presence of endogenous Mad1 and nocodazole. Representative images are at the *left* with quantifications at the *right*. Each dot represents the average kinetochore signal from a single cell; cells from three biological replicates are colored differently. Dunn's multiple comparisons test was used to determine *P*-values. Scale bars, 5 µm. All averages and error bars are median values and 95% confidence intervals.

Our initial validation focused on regions predicted by all three analysis methods, so next we validated a case where analysis methods showed less agreement: the *MAD1L1* gene. Consistent with previous literature, SURF, ProTiler, and TiVex all agreed that the C terminus of the protein is particularly important for its essential activity (Fig. 5A). This region is responsible for binding to kinetochore factors like Bub1 and Cdc20 (Brady and Hardwick 2000; Kim et al. 2012; London and Biggins 2014a; Allan et al. 2020; Fischer et al. 2021; Lara-Gonzalez et al. 2021; Piano et al. 2021). However, in the 600 amino acids upstream of that region we saw very little agreement between SURF, ProTiler, and TiVex (Fig. 5A). Using the same approach (Fig. 4A), we tested the ability of four mutant proteins with deletions outside the C-terminal region to complement *MAD1L1* knockout. Consistent with previous observations (Rodriguez-Bravo et al. 2014; Allan et al. 2020), the Mad1 protein was long lived and complementation assays could only be performed 10 d after Cas9:sgRNA transfection, resulting in greater variability for this assay. Nevertheless, Mad1^WT^ and Mad1^170Δ^ partially rescued the proliferation defect, while mutants Mad1^25Δ^ and Mad1^272Δ^ that were identified by SURF and TiVex did not, recapitulating screen results (Fig. 5B). Mad1^387Δ^, which was identified only by SURF, rescued viability but not reliably (Fig. 5B). The 10 d required to deplete Mad1 protein led to high variability in proliferation assays that would confound more nuanced mitotic phenotypes, so we further interrogated the biological functions of these essential regions in the presence of endogenous Mad1 protein, as has been done by others (Kim et al. 2012). We validated that none of the mutations compromised protein stability (Fig. 5C) and then determined whether highly expressed mutant proteins were able to perform an essential Mad1 activity: maintaining the spindle assembly checkpoint. We induced expression of each Mad1 protein overnight and then treated cells with the microtubule-destabilizing drug nocodazole for 20 h, which should trigger a robust SAC arrest. However, we found that fewer cells expressing Mad1^387Δ^ arrested in mitosis following this treatment, indicating that this region of Mad1 contributes to SAC signaling (Fig. 5D).

Robust SAC signaling requires that Mad1 localize to the kinetochore and further assemble the biochemical inhibitor of mitotic progression (Brady and Hardwick 2000; De Antoni et al. 2005; Lara-Gonzalez et al. 2021; Piano et al. 2021). Thus, we assayed the ability of mutant proteins to localize to kinetochores in cells either normally transiting mitosis or experiencing a robust SAC signal due to nocodazole treatment. We found that only the Mad1^387Δ^ protein exhibited kinetochore localization defects, which occurred specifically when cells were treated with nocodazole (Fig. 5E; Supplemental Fig. S6). In these cells, Mad1^387Δ^ kinetochore levels were reduced, yet a significant amount of protein still localized, indicating that at least one kinetochore recruitment mechanism remained functional in this mutant.

### Mad1^387Δ^ and Mad1^R617A^ contribute to kinetochore recruitment independently

Recent evidence suggests that Mad1 is initially recruited to kinetochores by the protein Bub1, but when kinetochores remain unattached to microtubules for long periods (such as in nocodazole), the RZZ complex (Rod, Zw10, and Zwilch) recruits a separate population of Mad1 to kinetochores (Kim et al. 2012; Silió et al. 2015; Zhang et al. 2015; Rodriguez-Rodriguez et al. 2018). We hypothesized that Mad1^387Δ^ contributes to an interaction with RZZ, which would explain the mixed results in the proliferation retest: Normally cycling cells should not rely on RZZ recruitment of Mad1, which is only required when chromosome alignment defects occur. This is also consistent with Mad1^387Δ^ localizing to prometaphase kinetochores, but not those arrested in nocodazole (Fig 5E; Supplemental Fig. S6).

**Figure 6.**
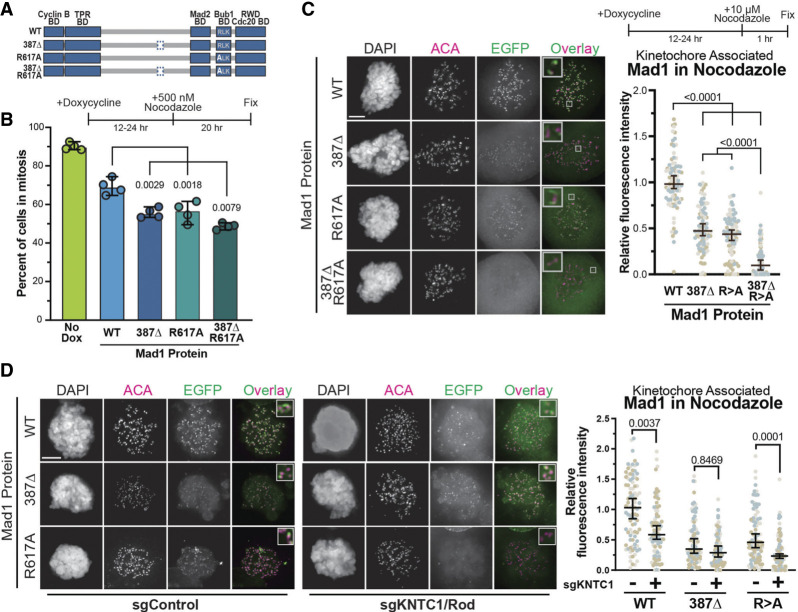
Mad1^387Δ^ contributes to kinetochore localization independent of the RLK motif that mediates Bub1 interaction. (*A*) Schematic of the Mad1^387Δ^ deletion mutant (removal of amino acids 387–402) and Mad1^R617A^ point mutant alone or in combination. (*B*) The ability of wild-type or mutant proteins to maintain a prolonged SAC arrest in the presence of endogenous Mad1 was assayed by treating cells with nocodazole for 20 h and determining the percent of cells in mitosis based on chromatin morphology. Data in “No Dox” were determined from all four cell lines not exposed to doxycycline. Each dot represents a biological replicate, and Dunn's multiple comparisons test was used to determine *P*-values. (*C*,*D*) Kinetochore association of EGFP-Mad1 wild-type and mutant fusion proteins was determined by the EGFP signal proximal to the anticentromere antibody (ACA) in the presence of endogenous Mad1 and nocodazole (*C*) and under the same conditions with KNTC1/Rod deleted (*D*). Representative images are at the *left* with quantifications at the *right*. Each dot represents the average kinetochore signal from a single cell; cells from three biological replicates are colored differently. Dunn's multiple comparisons test was used to determine *P*-values. Scale bars, 5 µm. All averages and error bars are median values and 95% confidence intervals.

Thus, to distinguish between the Bub1 and RZZ recruitment pathways, we also inhibited the well-characterized Bub1 binding “RLK motif” in Mad1 by mutating arginine 617 to alanine (Mad1^R617A^) and preventing its biochemical association with Bub1 (Brady and Hardwick 2000; Kim et al. 2012; Zhang et al. 2015; Fischer et al. 2021). We generated the Mad1^R617A^ mutant alone or in combination with Mad1^387Δ^ to determine whether mutating both regions entirely prevented kinetochore recruitment (Fig. 6A). We expressed these mutant proteins and found that fewer cells were able to maintain a SAC arrest in mitosis when expressing Mad1^387Δ^ or Mad1^R617A^ versus Mad1^WT^ (Fig. 6B). When the mutations were combined, we observed a slight additive effect, but we suspect this was limited by the presence of endogenous Mad1 protein (Fig. 6B). Consistent with the loss in SAC activity, we found that both mutations compromised Mad1 kinetochore association by ∼50% after 1 h of nocodazole treatment (Fig. 6C). When the mutations were combined, the protein virtually failed to localize to kinetochores. Consistent with previous results (Kim et al. 2012), this suggests that neither Mad1^387Δ^ nor Mad1^R617A^ dimerizes with endogenous protein or that such dimers fail to bind kinetochores. More importantly, this indicates that Mad1 residues 387–402 contribute to its kinetochore localization in a manner that is likely independent of the Bub1 interaction. To further dissect this, we electroporated cells encoding Mad1 proteins with a Cas9:sgRNA complex targeting RZZ member *KNTC1*/Rod and assayed Mad1 kinetochore recruitment 7 d later. We found that knockout of *KNTC1* (Supplemental Fig. S7) reduced the kinetochore localization of Mad1^WT^ and Mad1^R617A^ in nocodazole but had no effect on Mad1^387Δ^ (Fig. 6D). Thus, it is likely this region mediates or stabilizes an interaction with RZZ or another fibrous corona member.

To test this, we asked whether an endogenous RZZ member (Zw10) copurified with EGFP-tagged Mad1 proteins. Cells were synchronized by arresting them in S phase and releasing; then, as the population entered mitosis (based on cell rounding), cells were treated with nocodazole for 1 h. Surprisingly, immunopurifying either Mad1 or Zw10 in the different mutant backgrounds demonstrated no significant difference in the interaction between Zw10 and Mad1 proteins (Supplemental Fig. S8). Thus, it appears that Mad1^387Δ^ contributes to kinetochore localization through the RZZ pathway, but not by regulating their interaction in solution.

## Discussion

We have demonstrated that CRISPR–Cas9 tiling mutagenesis of endogenous protein-coding sequences in the human genome can be used to functionally validate and identify critical protein regions, including conserved and divergent protein sequences. Our approach takes advantage of the naturally occurring mutagenic properties of error-prone NHEJ in human cell lines after a dsDNA break is introduced by Cas9 activity.

In the process of validating CRISPR–Cas9 tiling as a discovery tool, we generated a powerful resource for the study of kinetochore genes, including a set of experimentally validated sgRNA sequences, but more importantly, 50–186 essential regions in 36 kinetochore proteins that have not yet been studied (Fig. 3B; Supplemental Table S2). Previous efforts to dissect human kinetochore factors relied on structure or sequence homology to guide truncations or mutations, but our functional screening was not limited in this way (Fig. 3D,E). Revealing important regions that would otherwise take years of laboratory work to identify expedites our collective molecular understanding of kinetochore biology and can be applied to other biological questions.

CRISPR–Cas9 tiling also enabled the unbiased discovery of an uncharacterized kinetochore localization motif in *MAD1L1*/Mad1. Mad1 localization to the kinetochore is dependent on interactions with Bub1 (Brady and Hardwick 2000; Kim et al. 2012; Silió et al. 2015; Lara-Gonzalez et al. 2021; Piano et al. 2021) and the RZZ complex (Kim et al. 2012; Zhang et al. 2015; Rodriguez-Rodriguez et al. 2018). Our findings are consistent with studies that indicate that two populations of Mad1 exist at the kinetochore and that they rely on distinct regulatory mechanisms (Kim et al. 2012; Zhang et al. 2015, 2019; Rodriguez-Rodriguez et al. 2018). However, our data suggest that this region of Mad1 does not contribute to the physical interaction with RZZ despite three-dimensional mapping of kinetochore organization placing the RZZ complex in direct proximity to Mad1 residues 387–402 (Roscioli et al. 2020). Instead, this region may cooperate with the N terminus of Mad1, which others have shown interacts with Cyclin B1 to facilitate corona localization. However, in our experiments, the Cyclin B1 interaction should be compromised due to the N-terminal EGFP tag (Allan et al. 2020). Altogether, this indicates that the RZZ-dependent recruitment of Mad1 to the kinetochore is a complex, likely multivalent, process, and hopefully our novel mutant will help uncover more of the mechanism in the future.

While powerful, our current tiling approach has some limitations. First, library coverage and domain resolution are partly determined by the “NGG” PAM, required by the type II CRISPR–Cas system from *Streptococcus pyogenes* (Mali et al. 2013). For example, we cannot make conclusions about regions where large gaps in library coverage exist (148-nt maximum spacing) due to a lack of NGG sequences (Fig. 1C). CRISPR nucleases with a more permissive PAM sequence (e.g., xCas9 or Cas9-NG) (Hu et al. 2018; Nishimasu et al. 2018) should enhance tiling screens by allowing more uniform and closer spacing between sgRNAs. Second, without modification, this approach will not identify regions for which a redundant gene exists (Fig. 2C). Third, while tiling mutagenesis appears robust when assaying aneuploid cell lines, gross genetic alterations (e.g., chromosome rearrangements, gene fusions, and SNPs) may confound analysis of some genes (Munoz et al. 2016). Despite these limitations, we found that essential gene regions are readily identified; and there are few if any false positives.

Altogether, this screening strategy is widely applicable, and the cost and scale of tiling libraries are magnitudes more reasonable than chemical- or UV-induced mutagenesis strategies in human cells. Similarly, tiling mutagenesis targets endogenous genomic loci, making it a better readout of cellular activity than libraries of mutant proteins expressed with highly active promoters from ectopic loci. Tiling mutagenesis screens are also an important advance beyond computational approaches that infer function based on sequence homology because tiling annotations are derived from phenotypic outcomes and thus ensure regions identified are truly important for protein function. Additionally, because sgRNA can be targeted nearly anywhere in this functional screen, important protein domains can be identified in regions resistant to homology-based analysis; namely, disordered protein regions and rapidly evolving sequences.

## Materials and methods

Key resources are available in Supplemental Table S4.

### Mammalian cell culture

HeLa, ARPE^TERT^, ARPE^RAS^, HCT116, 293T, and HeLa FlpIn (Taylor et al. 1998; Etemad et al. 2015) cells were grown in a high-glucose DMEM (Thermo Fisher Scientific 11-965-118/Gibco 11965118) supplemented with antibiotic/antimycotic (Thermo Fisher Scientific 15240062) and 10% fetal bovine serum (Thermo Fisher Scientific 26140095) at 37°C supplemented with 5% CO_2_. For microscopy experiments, cells were seeded in 35-mm wells containing acid-washed 1.5-mm × 22-mm square coverslips (Fisher Scientific 152222) and grown for 12–24 h prior to transfections or immunostaining; most treatments are outlined in the figures. The identity of each cell line was routinely validated by the presence of unique genetic modifications (Frt site, drug resistance genes, and expression of transgenes) to ensure cross-contamination did not occur. Cell lines were also regularly screened for mycoplasma contamination using DAPI staining. To entirely depolymerize the microtubule cytoskeleton prior to immunofluorescence staining, cells were treated with 10 µM nocodazole (Sigma-Aldrich M1404) for 1 h. To test SAC activity, cells were instead treated with 500 nM nocodazole (Sigma-Aldrich M1404) for 20 h prior to fixation. Cells were blocked in S phase by incubation with 250 µM thymidine for 16 h.

### Library cloning

A pooled single-stranded DNA 60-mer library containing all sgRNA sequences was synthesize by Twist Biosciences. Oligomers were designed with a universal 20 nt flanking the 5′ and 3′ with unique sgRNA sequences in the middle 20 nt. The library was PCR-amplified using universal primers that annealed to the common flanking sequence and appended homologous sequences at 5′ and 3′ ends of the PCR product to enable Gibson assembly (New England Biolabs E2611) into pZLCv2_puro_1KF. The vector pZLCv2_puro_1KF was linearized by digestion with restriction enzyme Esp3I, and both the PCR product and vector were gel-purified prior to assembly.

### CRISPR/Cas9 screening

Outgrowth screens were performed as previously described. The library of sgRNA-containing donor plasmids, pPAX2, and pMD2.G were cotransfected into 293T cells using polyethyleneimine (PEI; Polysciences 23966-1). Virus-containing supernatant media were harvested 48 h after transfection and passed through 0.45-µm filters, concentrated by centrifugation, and stored at −80°C. Each cell line was infected with varying volumes of concentrated virus in the presence of polybrene (Sigma Aldrich 107689) to determine the concentration that conferred survival in puromycin to 30% of cells, representing an MOI of 0.3, where a single infection per cell is the most likely outcome. Three replicates of each cell line were infected at scale to ensure 650× representation of the library and then 24 h later were exposed to 1 µg/mL puromycin. Seventy-two hours after infection, the puromycin-containing medium was replaced with drug-free medium. Ninety-six hours after infection, cells were trypsinized and reseeded to maintain 650× representation, while excess cells were harvested as an initial time point. Over the next 8 d, replicates were subcultured to maintain representation and eventually harvest a final population. Genomic DNA was extracted from 5 million cells (∼650× representation) in the initial and final populations, each using a QiaAMP DNA blood purification mini kit (Qiagen 51104), and then sgRNA sequences were amplified from each sample using a two-step PCR. For the first step, a 12-cycle PCR was performed using Phusion polymerase (New England Biolabs M0530) to amplify from all the genomic DNA extracted from the 5 million cells per sample (70–80 reactions). For the second step, an 18-cycle PCR was amplified from the pooled first step using primers coding 6-bp Illumina sequencing barcodes used for multiplexing biological samples. The final amplicon was purified from genomic DNA using a Monarch PCR and DNA cleanup kit (New England Biolabs T1030) and quantified with a Qubit 2.0 fluorometer. Samples were then sequenced using an Illumina HiSeq 2500. Deconvoluted sequencing results were submitted to NCBI's GEO repository under the submission record GSE179188 (https://www.ncbi.nlm.nih.gov/geo/query/acc.cgi?acc=GSE179188).

### Computational analysis of tiling data

Relative changes to the amount of sgRNA sequence detected in final versus initial samples were determined by the CRISPR–SURF package run from the command line (https://github.com/pinellolab/CRISPR-SURF) (Hsu et al. 2018). The SURF package includes “CRISPR–SURF Count,” which outputs logFC values for each sgRNA within the library. The logFC was calculated for each replicate, and the median value was used to generate a *Z*-score for each cell line. This output was used by CRISPR–SURF to deconvolve tiling data and identify the targeted genomic regions that had a negative effect on proliferation relative to nontargeting controls. Output *Z*-scores were also the input for ProTiler (https://github.com/MDhewei/protiler) (He et al. 2019) and TiVex. In a few instances, data were excluded from computational analysis. CRISPR–SURF Count did not report values for sgRNAs containing a TTTT repeat due to their likelihood of causing premature transcriptional termination. No other data were removed from global lists, but in the case of genes *BIRC5* and *KNL1*, we generated the library using transcripts containing rare or mutually exclusive exons, and when analyzing them at the protein level we mapped results to a more common transcript that does not contain those regions.

Tiling data with a convex fused lasso (TiVex) analysis built on previous approaches for analyzing tiling data that used a fused lasso to deconvolve complex signals. The fused lasso optimizes the cost function $arg\;\underset{x}{\min}\left(0.5*{\left\|y-x\right\|}^{2}+\lambda_{0}\left\|x\right\|+\lambda_{1}\left\|x_{i}-x_{i-1}\right\|\right)$ (Tibshirani and Taylor 2011; Hsu et al. 2018), but this was designed for sparse regulatory elements, while functional motifs in proteins are large blocks and may cover a large portion of proteins. If the sparsity-induced penalty is reduced (*λ*_0_ = 0), then the cost function is equivalent to identifying segmentations and not useful. To balance sparseness, we used a convex fused kasso (Parekh and Selesnick 2015) to deconvolve the data. This approach optimizes the cost function $\arg\;\underset{x}{\min}[0.5*{\left\|y-x\right\|}^{2}+\lambda_{0}\underset{i}{\sum }f(x_{i},\;a_{0})+\lambda_{1}\underset{i}{\sum }f(x_{i}-x_{i-1},\;a_{1})]\;$, where *f*(.) is a transform function, and 1 − *a*_0_*λ*_0_ − 4*a*_1_*λ*_1_ ≥ 0 defines the convex shape in the transformed space. TiVex regions were identified as negatively enriched by comparing the per-gene signal with a global average of all genes. Code for TiVex analysis will be made publicly available upon publication at https://labs.icahn.mssm.edu/zhulab/software or https://github.com/integrativenetworkbiology.

### Statistics

Outside of tiling analysis packages, GraphPad Prism version 9.1.0 was used for statistical analysis. Each test (paired, multiple comparison, etc.) is specifically identified in the figure legends, and generally error bars represent 95% confidence intervals.

### Generation of modified human cell lines

HeLa FlpIn Trex cells encoding wild-type and mutant proteins were generated as previously described (Herman et al. 2020). Briefly, HeLa FlpIn Trex cells were transfected with Flp recombinase (p0G44) and a donor plasmid encoding the protein of interest using Lipofectamine 2000 (Invitrogen 11668027) according to the manufacturer's instructions or PEI (Polysciences 23966-1). Forty-eight hours after transfection, the medium was supplemented with 500 µg/mL hygromycin (Invitrogen 10687010), and cells were negatively selected for 3 d. Expression of EGFP fusion proteins was then induced by addition of 1 µg/mL doxycycline (Sigma-Aldrich D9891), and EGFP-expressing cells were positively selected by FACS. Doubly selected polyclonal populations were frozen and stored for future experiments.

### Nucleic acid reagents

Mad1 and Sgo1 FRT/TO/Hygro vectors were a gift from Jennifer DeLuca. The coding DNA sequences for Mad1 and Sgo1 were amplified from cDNA libraries and thus, for proliferation retests, synthetic sgRNA targeting these genes spanned intron–exon boundaries to ensure the ectopic copy was not targeted. All other coding sequences were generated as codon-optimized and thus sgRNA-resistant gBlocks (IDT), inserted into restriction enzyme linearized pcDNA5 FRT/TO/Hygro by Gibson assembly, and sequence-verified.

### sgRNA:Cas9-mediated gene knockout

Genes were knocked out using one to two synthetic sgRNAs (Synthego) in complex with spCas9 (Aldeveron 9214) that were electroporated into cells using a nucleofector system (Lonza V4XC-1032) according to published methods (Hoellerbauer et al. 2020a,b). Briefly, 120,000 cells were mixed with either targeting or nontargeting sgRNA:Cas9 complexes in complete SE nucleofector solution. Cell solutions were added to 16-well minicuvette cells and electroporated using program CN-114. Cells were split into two wells, one with doxycycline and one without, and cell numbers were assayed 5–10 d later. KNTC1 knockout by RNP electroporation was validated by analyzing gDNA 7 d after electroporation. gDNA was extracted, and the target locus was amplified by PCR, Sanger-sequenced, and deconvolved using ICE (Synthego).

### Immunoblotting

Expression of Flag- and EGFP-tagged proteins was induced with media containing 1 µg/mL doxycycline (Sigma-Aldrich D9891) 12–24 h prior to harvesting. Cells were isolated via trypsinization and then centrifuged. Immunoblotting was performed as previously described (Herman et al. 2020). Trypsinized cells were then resuspended in complete lysis buffer and frozen in liquid nitrogen. Samples were thawed and sonicated with a CL-18 microtip for 20 sec at 50% maximum power with no pulsing three times using a Fisher Scientific FB50 sonicator. Benzonase nuclease (Millipore E1014) was added to samples and incubated for 5 min at room temperature, and then samples were centrifuged at 16,100*g* at 4°C in a tabletop centrifuge. Relative protein concentrations were determined for clarified lysates, and samples were normalized through dilution. Denatured samples were run on Tris-buffered 10% or 12% polyacrylamide gels in a standard Tris-glycine buffer. Proteins were transferred to a 0.45-µm nitrocellulose membrane (Bio-Rad 1620115) for 2 h at 4°C in a transfer buffer containing 20% methanol. Membranes were washed in PBS + 0.05% Tween-20 (PBS-T), blocked with PBS-T + 5% nonfat milk, and incubated with primary antibodies overnight at 4°C. Antibodies were diluted in PBS-T by the following factors or to the following concentrations: 1 µg/mL anti-GAPDH clone 6C5 (Millipore Sigma MAB374), 0.5 µg/mL anti-GFP clone JL-8 (Takara 632381), 2 µg/mL anti-Flag clone M2 (Sigma Aldrich F3165), and 1:1000 anti-Zw10 (ProteinTech 24561-1-AP). HRP-conjugated antimouse and antirabbit secondary antibodies (GE Lifesciences NA931 and NA934) were diluted 1:10,000 in PBS-T and incubated on membranes for 45 min at room temperature. Immunoblots were developed with enhanced chemiluminescence HRP substrate SuperSignal West Dura (Thermo Scientific 34076) using a ChemiDoc MP system (Bio-Rad).

### Immunofluorescent staining

Upon completion of experimental manipulations, cells grown on coverslips were immediately chemically cross-linked for 15 min with 4% PFA diluted from a 16% stock solution (Electron Microscopy Sciences 15710) with 1× PHEM (60 mM PIPES, 25 mM HEPES, 5 mM EGTA, 8 mM MgSO_4_) or 1× PHEM + 0.5% Triton X-100. Coverslips were washed with 1× PHEM + 0.5% Triton X-100 for 5 min and then washed three more times with 1× PHEM + 0.1% Triton X-100 over 10 min. Cells were blocked for 1–2 h at room temperature in 20% goat serum in 1× PHEM. Anticentromere protein antibody or ACA (Antibodies, Inc. 15-235) and anti-Rod antibody (Santa Cruz Biotechnology sc81853) were diluted in 20% goat serum at a 1:600 and 1:100 dilution factor, respectively. Coverslips were incubated overnight at 4°C in the primary antibody and then washed four times with 1× PHEM + 0.1% Triton X-100 over 10 min. Goat antihuman secondary antibodies conjugated to Alexa Fluor 647 (Invitrogen) and goat antimouse secondary antibodies conjugated to Alexa Fluor 568 (Invitrogen) were diluted at 1:300 in 20% boiled goat serum. Coverslips were washed four times with 1× PHEM + 0.1% Triton X-100 over 10 min and then stained for 1 min with 30 ng/mL 4′,6-diamidino-2-phenylindole (DAPI; Invitrogen D1306) in 1× PHEM. Coverslips were washed twice with 1× PHEM, immersed in mounting medium (90% glycerol, 20 mM Tris at pH 8.0, 0.5% [w/v] N-propyl gallate) on microscope slides, and sealed with nail polish.

### Microscopy and image analysis

Fixed cell images were acquired on a DeltaVision Ultra deconvolution high-resolution microscope (GE Healthcare) equipped with a 60×/1.42 PlanApo N oil immersion objective (Olympus) and a 16-bit sCMOS detector. Cells were imaged in Z-stacks through the entire cell using 0.2-µm steps. All images were deconvolved using standard settings. Z projections of the maximum signal in all channels were exported as TIFFs for analysis by Cell Profiler 4.0.7 (29969450). ACA images were used to identify regions of interest after using a global threshold to remove background signal and distinguishing clumped objects using signal intensity. The signal intensity within these regions was quantified from all other images, and then for background correction the regions were expanded by one pixel along the circumference, and signal intensity was again quantified in the appropriate channel. Background intensity was found by subtracting the intensity of the original region from the one-pixel expanded region. The background intensity per pixel was quantified by dividing the background intensity by the difference in area between two regions. This was then multiplied by the area of the original object and subtracted from the intensity of the original object, and any negative values were changed to zero. The mean value per image was then determined and is displayed in the figures. Representative images displayed from these experiments are projections of the maximum pixel intensity across all Z images. Photoshop was used to crop; make equivalent, linear adjustments to brightness and contrast; and overlay images from different channels.

## Supplementary Material

Supplemental Material
