# Supplementary material for: Functional dissection of human mitotic genes using CRISPR–Cas9 tiling screens
Source: Genes Dev. 2022 Apr 1;36(7-8):495–510. doi: 10.1101/gad.349319.121 (PMC9067404; doi:10.1101/gad.349319.121)
Supplement: Supplemental Material [file supp_36_7-8_495__DC1.html]

Functional dissection of human mitotic genes using CRISPR–Cas9 tiling screens — Supplemental Material 

# Functional dissection of human mitotic genes using CRISPR–Cas9 tiling screens

## Supplemental Material

- Supplementary\_Table1.xlsx
- Supplementary\_Table2.xlsx
- Supplementary\_Table3.xlsx
- Supplementary\_Table4.xlsx
- Supplemental\_Material.pdf
