# Supplementary material for: Functional dissection of human mitotic genes using CRISPR–Cas9 tiling screens
Source: Genes Dev. 2022 Apr 1;36(7-8):495–510. doi: 10.1101/gad.349319.121 (PMC9067404; doi:10.1101/gad.349319.121)
Supplement: Supplemental Material [file supp_gad.349319.121_Supplemental_Material.pdf]

Description of Supplementary Tables

Supplementary Table 1: sgRNA sequences, targets, raw, and normalized counts from Illumina sequencing.
Supplementary Table 2: Tiling regions identified using CRISPR SURF, ProTiler, and TiVex in 48 kinetochore genes
Supplementary Table 3: Annotations within 48 kinetochore genes from D2P2 and Pfam databases and manually curated from published literature.
Supplementary Table 4: Key resources used for this study.

Supplementary Methods

Library Design

All possible sgRNA target sequences within the protein coding DNA sequence (CDS) of 48 target genes were identified using the Broad Institute's GPP sgRNA Designer (since redesigned as CRISPick) by inputting gene symbols (Doench et al. 2016; Sanson et al. 2018). CRISPick output sequences for every unique spCas9 PAM (NGG) which cut within the CDS of the consensus or longest transcript for each gene. An initial library was generated from sgRNA that uniquely targeted the gene of interest and no other exonic regions within the genome. To prevent bias in the library near poly-G repeats, sgRNA were removed to ensure a minimum spacing of 5 nt between cut sites within the CDS. In regions where there were >50 nts between sgRNA, we included sequences with limited off-target sites to increase the resolution of screening. One exception being last minute additions, CENP-S and CENP-X where all unique sgRNA were included with no adjustment for minimum or maximum spacing between sgRNAs.

Computational analysis of tiling data

We used CRISPR SURF and TiVex to identify protein domains in our datasets consisting of 4 different cell lines. The domains identified were called “Positive Domains”. To calculate “Negative Domains“, we divided up the regions which were not identified as a Positive Domain by CRISPR SURF or TiVex into 3 or 45 AA windows respectively for the 48 genes that were tiled. Next, we simulated datasets with low efficiency sgRNAs by randomly transforming x% (10, 20, 30, 40, and 50%) of sgRNAs targeting a gene from their true value to a NTC value. This was performed 10 times at each % resulting in 50 datasets. For each of these datasets, we used CRISPR-SURF or TiVex to identify protein domains. Domains of min width of 3 AA and FDR < 0.05 were considered in our analysis. For each percentage of signal lost, we pooled results across 10 runs and calculated True positive (TP), False Positive (FP), True Negative (TN) , False Negative (FN), Precision, Recall and F1 score. True Positives (TP) is the number of domains retrieved from simulated data that overlapped with a “Positive domain”. True Negative (TN) is the number of negative domains which were correctly classified as a negative domain in the simulated data and were also a “Negative domain”. False Positive (FP) is the number of domains that were identified when low efficiency sgRNAs were simulated but were a “Negative domain” in the original dataset. False Negative represents the positive domains which were not identified in the simulation. Precision is the fraction of correct domains among the total predicted domains calculated as: Precision =(TP)/(TP+FP). Recall is the fraction of correct domains among the total domains in the dataset. It indicates how many total domains of the actual dataset were picked up as a domain with noise and is

calculated as Recall =(TP)/(TP+FN). F1 score integrates Precision and recall and is calculated as F1 score = 2* ((precision*recall)/(precision+recall)).

Generation of Modified Human Cell Lines

RPE1 cells have been used to study chromosome segregation however they harbor genes conferring resistance to both hygromycin and puromycin making them incompatible with our screening approach. Thus, an alternative retinal pigment epithelial cell line, ARPE19, was purchased (ATCC CRL-2302) and immortalized using lentiviral transduction of hTert (Addgene 114316) and selected using 5 μg/mL blasticidin to generate $ARPE^{TERT}$. $ARPE^{RAS}$ were generated by viral transduction of numerous transgenes to enable expression of oncogenic $HRAS^{G12V}$ as previously described. (Kendall et al. 2005; Toledo et al. 2014).

Immunopurification

T-225 flasks of $Mad1^{WT}$, $Mad1^{\Delta 387}$, $Mad1^{R617A}$, and $Mad1^{\Delta 387+R617A}$ HeLa FlpIn Trex cells were grown to 50% confluence and arrested in S phase for 16 hours with 2.5 μM thymidine, then released via three PBS washes into drug free media. Eight hours later cells were arrested again in S phase for 16 hours in the presence of doxycycline to induce expression of Mad1 proteins. Cells were then washed three times with PBS and released into drug-free media. Four hours later as cells entered mitosis they were treated with 5 μM nocodazole for one hour. Cells were harvested by trypsin digestion, counted by hemocytometer, and then centrifuged. The cell pellet was resuspended in 1 μL of complete lysis buffer (25 mM HEPES, 2 mM MgCl2, 0.1 mM EDTA, 0.5 mM EGTA, 15%

Glycerol, 0.1% NP-40, 150 mM KCl, 1 mM PMSF, 1 mM sodium pyrophosphate, 1x Pierce Protease Inhibitor Cocktail [Thermo Scientific 88666]) for each 200,000 cells, and then snap frozen in liquid nitrogen. Samples were thawed and sonicated with a CL- 18 microtip for 15 s at 50% maximum power with no pulsing two times using a Fisher Scientific FB50 sonicator. Approximately 150 U of Benzonase nuclease (Millipore E1014) was added to samples and incubated at room temperature for 15 min. The samples were centrifuged at 16,100 x g at 4ºC in a tabletop centrifuge for 30 minutes. Clarified lysates were moved to fresh microfuge tubes and 60 mL of Protein G Dynabeads (Thermo Fisher Scientific 10009D) conjugated with anti-EGFP monoclonal antibody (Sigma Aldrich 11814460001) or anti-Zw10 polyclonal antibody (Proteintech 24561-1-AP) as previously described (Akiyoshi et al., 2009) were added to 250 µL of lysate and incubated at 4ºC with rotation for 90 min. Beads were washed six times with lysis buffer lacking PMSF, sodium pyrophosphate, and protease inhibitor cocktail. Proteins were eluted from beads in 60 mL of 1x SDS sample buffer and incubated at 95ºC for 10 minutes.

<u>Computational Analysis of Protein Sequences</u>

Databases of Conserved and Disordered Proteins

PhyloP scores for nucleotides within the genomic regions corresponding to essential regions identified by tiling were downloaded manually from UCSC genome browser and mean values calculated. Disordered regions were identified using the D2P2 database(Oates et al. 2013) based on the agreement of >75% of disorder prediction algorithms. Our comprehensive list of Pfam domains were identified using gene IDs.

Supplementary Figure Legends

Figure S1. Tiling library design strategy benefits from a lack of positional repair bias. (A) Calculations showing 36% of a CRISPR edited population should have at least one in-frame but mutagenized allele on average. (B) Scheme for choosing sgRNA included in the library. (C-D) All sgRNAs in the library were binned based on the position they target (% of CDS). (C) For each bin, in each gene, the percent of sgRNA predicted to cause 1nt indels based on sequence constraints (Chakrabarti et al. 2019) are plotted. (D) For each bin, in each gene, the mean percent likelihood of forming a frameshift mutation based on sequence constraints (Shen et al. 2018) are plotted. All violin plots show median (solid lines), quartiles (dotted lines) and range. (E-F) The standard deviation across each bin in (C) and (D) for each gene was calculated and is plotted as a single dot. Deviations are plotted relative to the CDS length (E) or the total number of sgRNAs targeting the gene (F). Data are fitted with a single-phase exponential decay curve.

Figure S2. Tiling library is reproducible and differences from genome-wide data have biological causes. (A) Each sgRNA's Z score from $ARPE^{TERT}$, $ARPE^{RAS}$, and HCT116 cells are plotted relative to each sgRNA's Z score in HeLa cells. These data exclude non-targeting controls and only show sgRNA in the bottom quartile of Z-scores from HeLa cells. The dashed line (y = x) is plotted for reference. (B) Correlation matrix and heatmap for the bottom quartile of targeting sgRNA in each cell line with Pearson correlation coefficients displayed. (C) Percent of sgRNAs with Z < -1.0 targeting each gene are shown for all four cell lines. Cell lines meeting the >8% cutoff are highlighted in teal. (D) Z scores for each sgRNA targeting genes where disagreement between tiling

and DepMap data disagree (*MIS12* or *MAD1L1*), with sgRNA sequences present in both libraries colored teal. (E) Numerous transcript models of *CENPM* and *SKA2* (right) along with their relative expression in cultured cells from public RNA-seq datasets (right). The location targeted by each sgRNA in the DepMap (Avana) library shown as teal bars indicating they target rarely used exons.

Figure S3. More evidence of SURF, ProTiler, and TiVex resolution. (A) Tiling profile of *KIF18A*, pink rectangles identify key motifs within the motor domain (ATP binding pocket and neck linker). (B) Tiling profile for *CKAP5*/chTOG (C) Crystal structure of TOG2 (~aa 250-500) from budding yeast homolog bound to a tubulin dimer (PDB: 4U3J) are colored based on the homologous regions identified by TiVex (green) and SURF (blue) as important for proliferation (Ayaz et al. 2014). Four residues absolutely required for the protein-protein interaction are shown in blowups. (D) Percent of protein coding sequence identified as essential by each analysis method is shown in comparison to boundaries identified in PFAM or literature. Each dot represents an individual gene and bars represent median values. (E) Score of the average conservation of nucleotides among 100 vertebrate species is grouped based on if they fall in tiling identified regions (essential) or those not recognized as significant by tiling (non-essential). (F) The number of regions identified per gene by each analysis method. Specific genes showing key differences between SURF and ProTiler are colored uniquely in all three columns.

Figure S4. Simulation to determine how low efficiency sgRNA affect tiling analysis. (A) Schematic showing how datasets simulating 10, 20, 30, 40, or 50% low efficiency sgRNAs were generated for all 48 mitotic factors, which were analyzed 10 times

each using CRISPR-SURF or TiVex. (B) Table representing the ability of simulated data to recapitulate original findings of CRISPR SURF analysis among all kinetochore genes, where 'positives' are identified in 8/10 simulations. (C) Box and whisker plot showing how often individual SURF regions were identified datasets simulating low efficiency sgRNAs. (D,E) Same analysis as B and C but for TiVex. Boxes in C and E represent median and quartiles while whiskers show range of datapoints.

Figure S5. Functional validation and characterization of 11 high-resolution regions within 5 genes identified by tiling, continued. (A) Average PhyloP scores across 100 vertebrates for each codon within deletion mutants from figures 4 and S5 are plotted. Dashed lines denote sequences statistically ($P<0.05$) unchanged (PhyloP > 1.3) or rapidly changing (PhyloP < -1.3) sequences. Boxes represent median and quartiles while whiskers show range of codons. (B-C) Tiling profile, validation of proliferation phenotype, and assay of protein stability for (B) CENPH/Cenp-H and (C) SGO1/Sgo1. All data are the same as in Fig. 3.

Figure S6. Mutations in Mad1 do not affect localization to kinetochores immediately following nuclear envelope breakdown. Kinetochore association of EGFP-Mad1 wild type and mutant fusion proteins was determined by the EGFP proximal to anti-centromere antibody (ACA) in the presence of endogenous Mad1 immediately after nuclear envelope breakdown. Representative images on left with quantifications on right. Each dot represents the average kinetochore signal from a single cell, cells from three biological replicates are colored differently. Dunn's multiple comparisons test was used to determine

P-values. Scale bars are 5 µm, all averages and error bars in figure are median values and 95% confidence intervals.

Figure S7. Electroporation of Cas9:sgKNTC1 RNPs causes robust deletion of KNTC1. (A) Kinetochore association *KNTC1*/Rod protein after knock out in cells from Figure 6 was determined by immunostaining for Rod and anti-centromere antibody (ACA). Representative images on left with quantifications on right. Each dot represents the average kinetochore signal from a single cell, cells from three biological replicates are colored differently. Dunn's multiple comparisons test was used to determine P-values. Scale bars are 5 µm, all averages and error bars in figure are median values and 95% confidence intervals. (B) Depiction of genomic site targeted by sgKTNC1 and primers used to validate deletion. (C) Sanger sequencing traces of PCR product from (B) in cells treated with sgNonTargetingControl (top) or sgKTNC1 (bottom). Edited sequences are aligned to the control population using sequence upstream of sgRNA target site (underlined sequence), which allows deconvolution of edited traces. (D) Deconvolution sequencing (B,C) was used to analyze cells treated with sgKNTC1 seven days after electroporation and percent of reads with frameshifting mutations is reported. Bars represent a mean value and error bars are 95% confidence intervals.

Figure S8. Mad1 mutants do not affect soluble interaction with Zw10. Immunoblots showing co-immunopurification of Mad1 proteins and RZZ complex member, Zw10. The same cell lysates (left) were used to purify exogenous EGFP-Mad1 (middle) or endogenous Zw10 (right) and then probe for GFP, Zw10, and GAPDH.

REFERENCES

Ayaz P, Munyoki S, Geyer EA, Piedra FA, Vu ES, Bromberg R, Otwinowski Z, Grishin NV, Brautigam CA, Rice LM. 2014. A tethered delivery mechanism explains the catalytic action of a microtubule polymerase. *eLife* **3**: e03069.

Chakrabarti AM, Henser-Brownhill T, Monserrat J, Poetsch AR, Luscombe NM, Scaffidi P. 2019. Target-Specific Precision of CRISPR-Mediated Genome Editing. *Mol Cell* **73**: 699-713 e696.

Doench JG, Fusi N, Sullender M, Hegde M, Vaimberg EW, Donovan KF, Smith I, Tothova Z, Wilen C, Orchard R et al. 2016. Optimized sgRNA design to maximize activity and minimize off-target effects of CRISPR-Cas9. *Nat Biotechnol* **34**: 184-191.

Kendall SD, Linardic CM, Adam SJ, Counter CM. 2005. A network of genetic events sufficient to convert normal human cells to a tumorigenic state. *Cancer Res* **65**: 9824-9828.

Oates ME, Romero P, Ishida T, Ghalwash M, Mizianty MJ, Xue B, Dosztanyi Z, Uversky VN, Obradovic Z, Kurgan L et al. 2013. D(2)P(2): database of disordered protein predictions. *Nucleic Acids Res* **41**: D508-516.

Sanson KR, Hanna RE, Hegde M, Donovan KF, Strand C, Sullender ME, Vaimberg EW, Goodale A, Root DE, Piccioni F et al. 2018. Optimized libraries for CRISPR-Cas9 genetic screens with multiple modalities. *Nature communications* **9**: 5416.

Shen MW, Arbab M, Hsu JY, Worstell D, Culbertson SJ, Krabbe O, Cassa CA, Liu DR, Gifford DK, Sherwood RI. 2018. Predictable and precise template-free CRISPR editing of pathogenic variants. *Nature* **563**: 646-651.

Toledo CM, Herman JA, Olsen JB, Ding Y, Corrin P, Girard EJ, Olson JM, Emili A, DeLuca JG, Paddison PJ. 2014. BuGZ is required for Bub3 stability, Bub1 kinetochore function, and chromosome alignment. *Dev Cell* **28**: 282-294.

Supplementary Figure 1

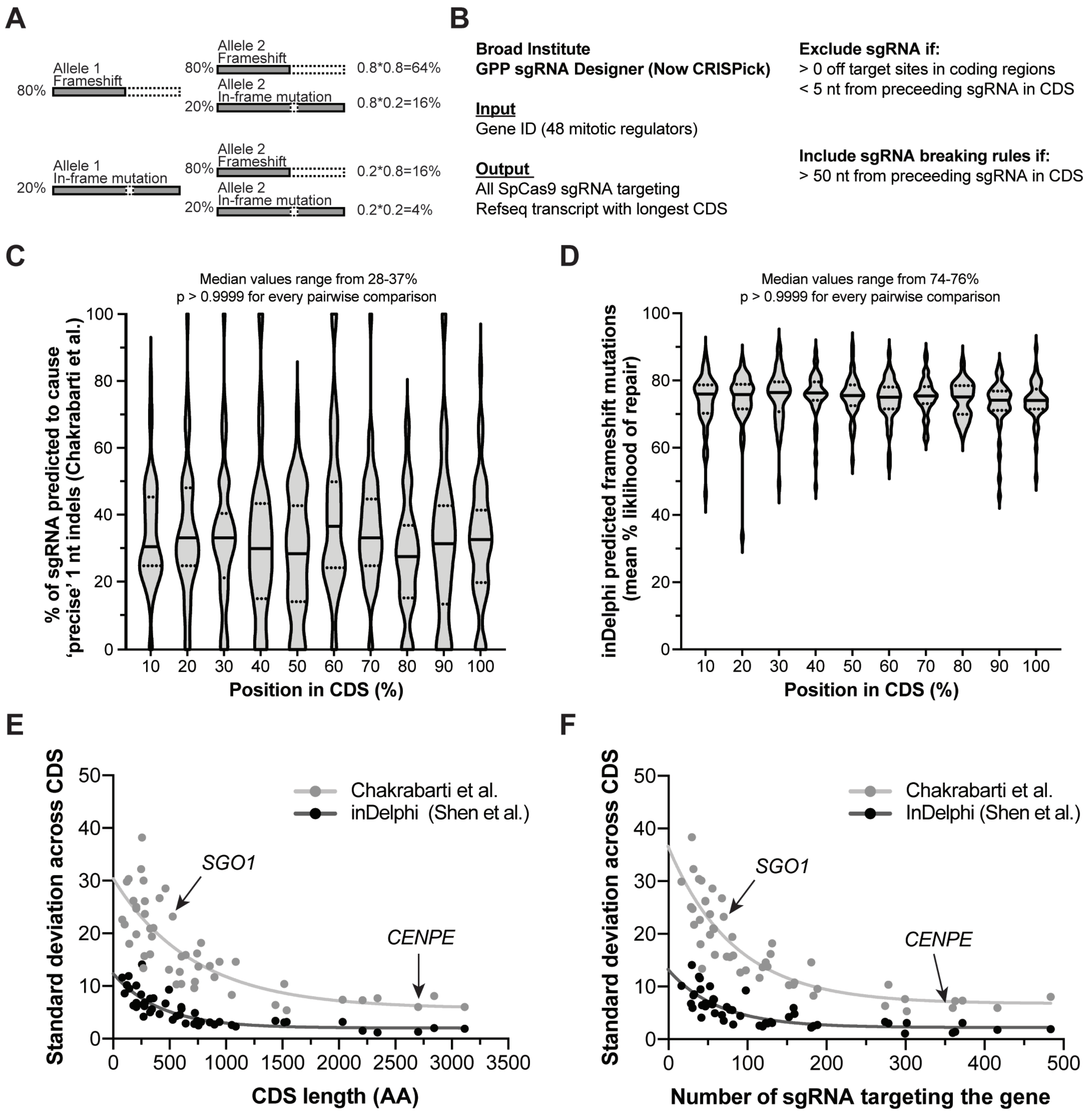

Figure S1. Tiling library design strategy benefits from a lack of positional repair bias. (A) Calculations showing 36% of a CRISPR edited population should have at least one in-frame but mutagenized allele on average. (B) Scheme for choosing sgRNA included in the library. (C-D) All sgRNAs in the library were binned based on the position they target (% of CDS). (C) For each bin, in each gene, the percent of sgRNA predicted to cause 1nt indels based on sequence constraints (Chakrabarti et al. 2019) are plotted. Violin plots show median (solid lines), quartiles (dotted lines) and range of bins for all genes. (D) For each bin, in each gene, the mean percent likelihood of forming a frameshift mutation based on sequence constraints (Shen et al. 2018) are plotted. Violin plots show median (solid lines), quartiles (dotted lines) and range. (E-F) The standard deviation across each bin in (C) and (D) for each gene was calculated and is plotted as a single dot. Deviations are plotted relative to the CDS length (E) or the total number of sgRNAs targeting the gene (F). Data are fitted with a single-phase exponential decay curve.

Supplementary Figure 2

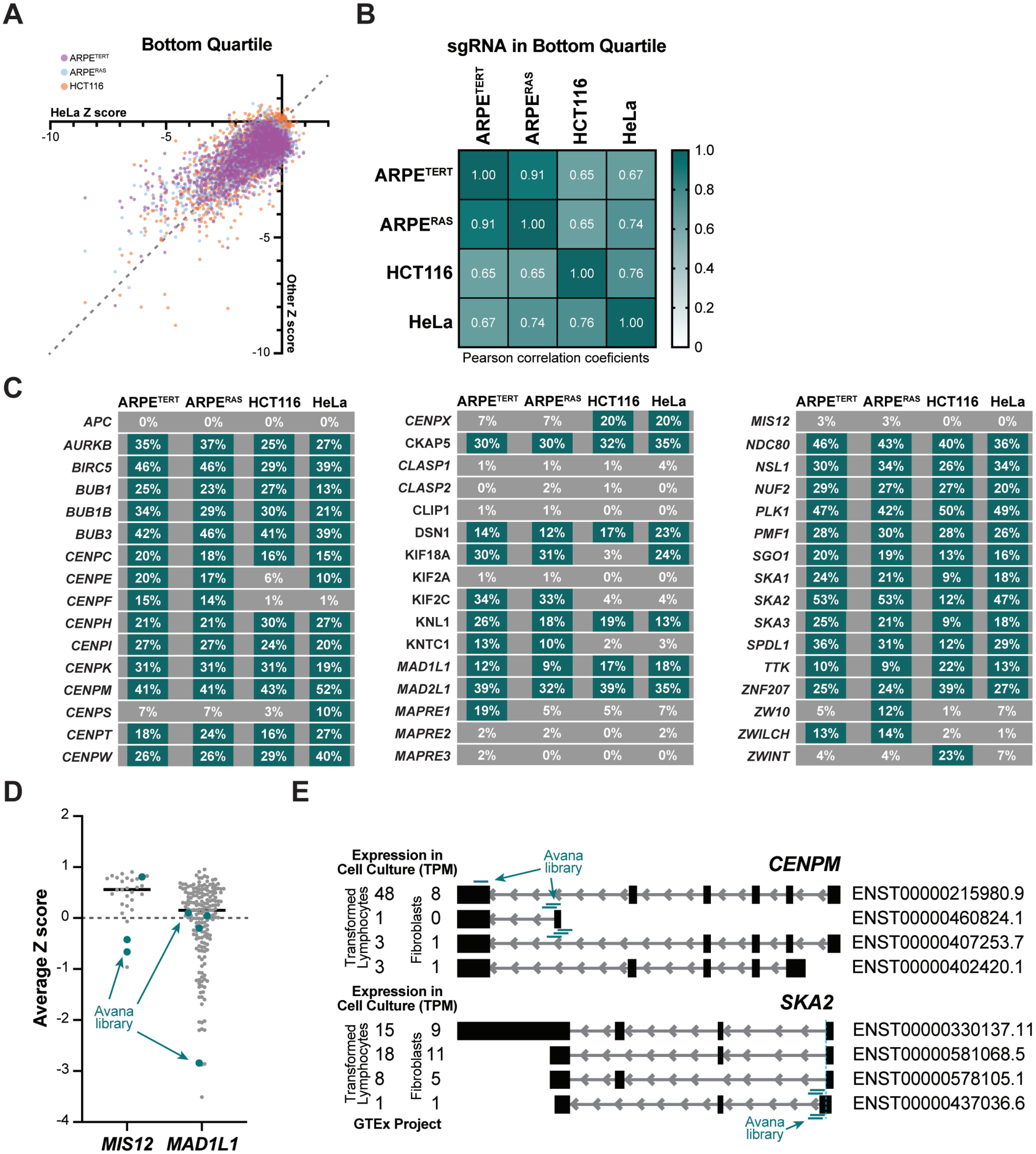

Figure S2. Tiling library is reproducible and differences from genome-wide data have biological causes. (A) Each sgRNA's average Z score from three replicates of ARPETERT, ARPERAS, and HCT116 cells are plotted relative to each sgRNA's average Z score from three replicates of HeLa cells. These data exclude non-targeting controls and only show sgRNA in the bottom quartile of Z-scores from HeLa cells. The dashed line (y = x) is plotted for reference. (B) Correlation matrix and heatmap for the bottom quartile of targeting sgRNA in each cell line with Pearson correlation coefficients displayed. (C) Percent of sgRNA with Z < -1.0 targeting each gene are shown for all four cell lines. Cell lines meeting the >8% cutoff are highlighted in teal. (D) Z scores for each sgRNA targeting genes where disagreement between tiling and DepMap data disagree (MIS12 or MAD1L1), with sgRNA sequences present in both libraries colored teal. (E) Numerous transcript models of CENPM and SKA2 (right) along with their relative expression in cultured cells from public RNA-seq datasets (right). The location targeted by each sgRNA in the DepMap (Avana) library shown as teal bars indicating they target rarely used exons.

Supplementary Figure 3

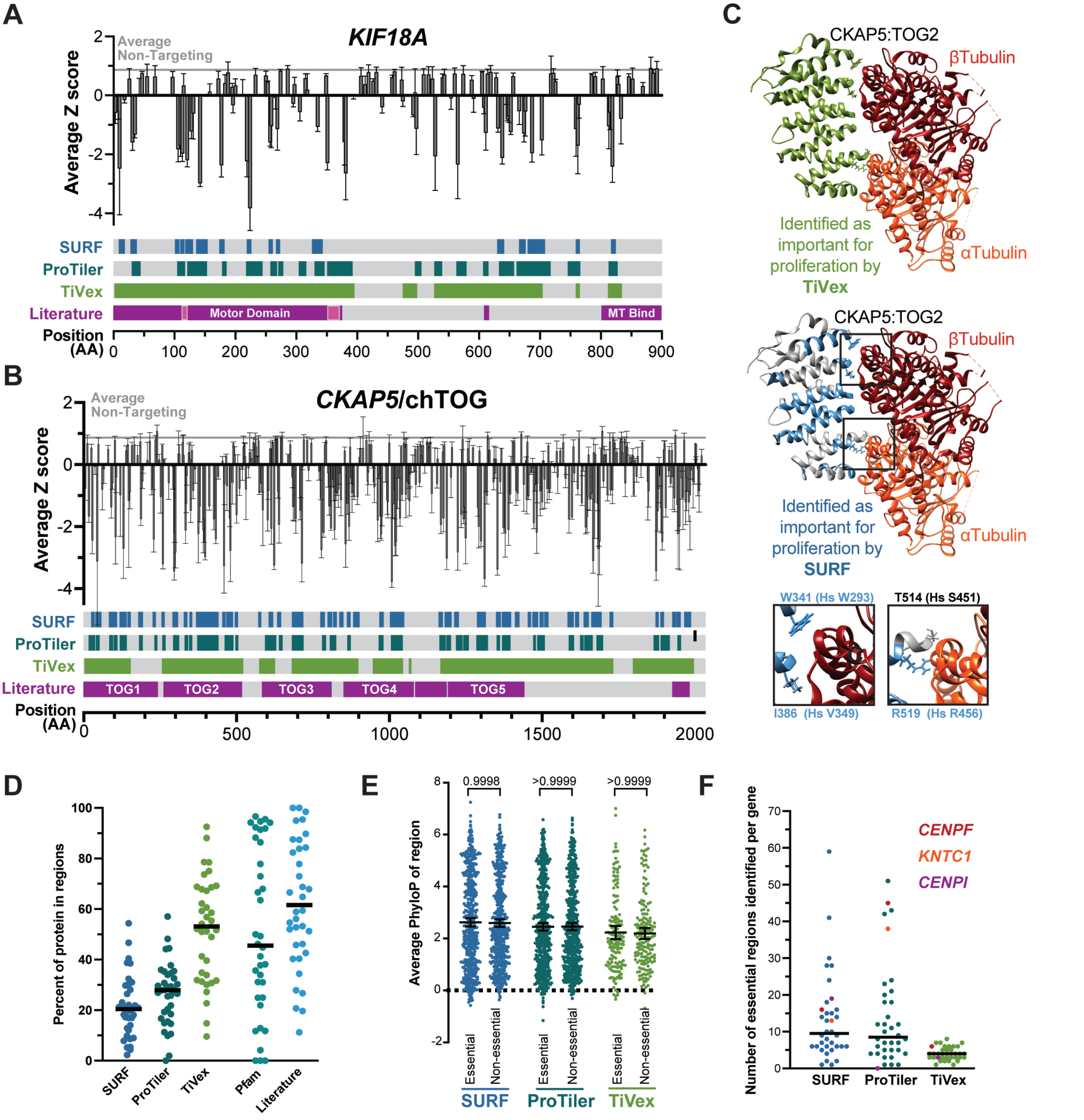

Figure S3. More evidence of SURF, ProTiler, and TiVex resolution. (A) Tiling profile of *KIF18A*, pink rectangles identify key motifs within the motor domain (ATP binding pocket and neck linker). (B) Tiling profile for *CKAP5*/chTOG (C) Crystal structure of TOG2 (~aa 250-500) from budding yeast homolog bound to a tubulin dimer (PDB: 4U3J) are colored based on the homologous regions identified by TiVex (green) and SURF (blue) as important for proliferation (Ayaz et al. 2014). Four residues absolutely required for the protein-protein interaction are shown in blowups. (D) Percent of protein coding sequence identified as essential by each analysis method is shown in comparison to boundaries identified in PFAM or literature. Each dot represents an individual gene and bars represent median values. (E) Score of the average conservation of nucleotides among 100 vertebrate species is grouped based on if they fall in tiling identified regions (essential) or those not recognized as significant by tiling (non-essential). (F) The number of regions identified per gene by each analysis method. Specific genes showing key differences between SURF and ProTiler are colored uniquely in all three columns.

Supplementary Figure 4

A

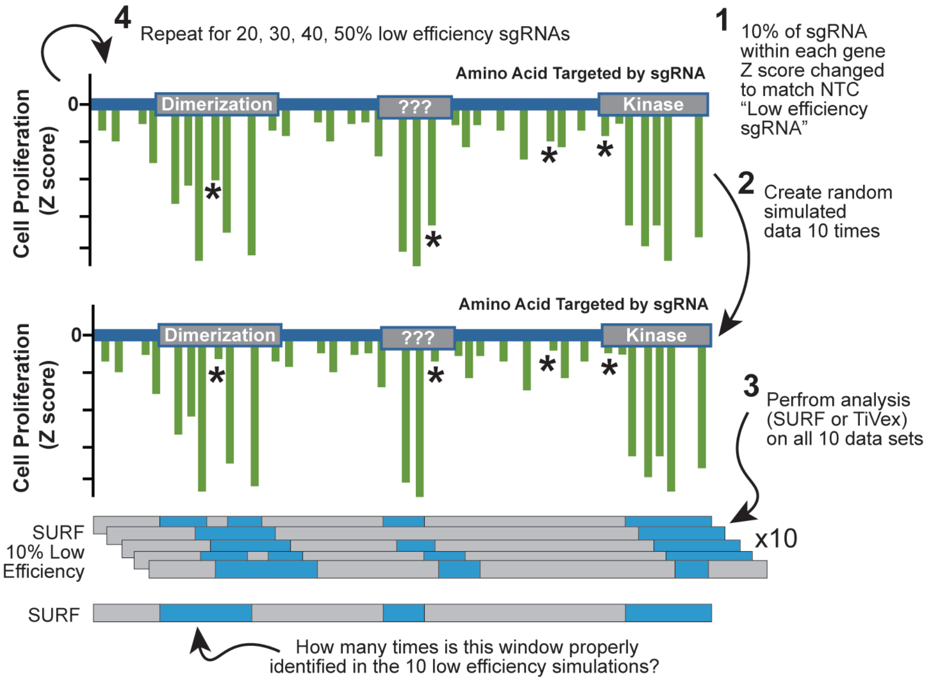

B

Regions identified 8/10 times	CRISPR-SURF 10%	20%	30%	40%	50%
True Positive	452	319	213	133	80
False Negative	141	274	380	460	513
True Negative	10172	10133	10126	10150	10138
False Positive	43	82	89	65	77
Precision	0.91	0.80	0.71	0.67	0.51
Recall	0.76	0.54	0.36	0.22	0.13
F1 Score	0.83	0.64	0.48	0.34	0.21

C

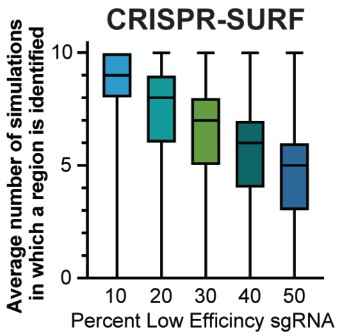

D

Regions identified 8/10 times	TiVex 10%	20%	30%	40%	50%
True Positive	110	109	80	70	54
False Negative	37	38	67	77	93
True Negative	1906	1913	1919	1921	1923
False Positive	22	15	9	7	5
Precision	0.83	0.88	0.90	0.91	0.92
Recall	0.75	0.74	0.54	0.48	0.37
F1 Score	0.79	0.80	0.68	0.63	0.52

Precision: $\frac{\text{True Pos.}}{\text{True Pos.+False Pos.}}$

Recall: $\frac{\text{True Pos.}}{\text{True Pos.+False Neg.}}$

F1 Score: $\frac{\text{Precision x Recall}}{\text{Precision + Recall}}$

E

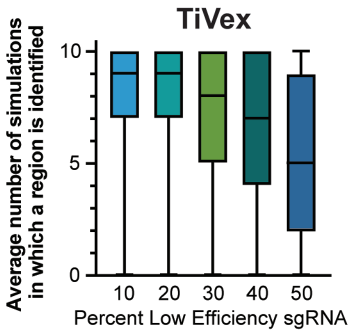

Figure S4. Simulation to determine how low efficiency sgRNA affect tiling analysis. (A) Schematic showing how datasets simulating 10, 20, 30, 40, or 50% low efficiency sgRNAs were generated and analyzed 10 times each using CRISPR-SURF or TiVex. (B) Table representing the ability of simulated data to recapitulate original findings of CRISPR SURF analysis among all kinetochore genes, where 'positives' are identified in 8/10 simulations. (C) Box and whisker plot showing how often individual SURF regions were identified datasets simulating low efficiency sgRNAs. (D,E) Same analysis as B and C but for TiVex. Boxes in C and E represent median and quartiles while whiskers show range of datapoints.

Supplementary Figure 5

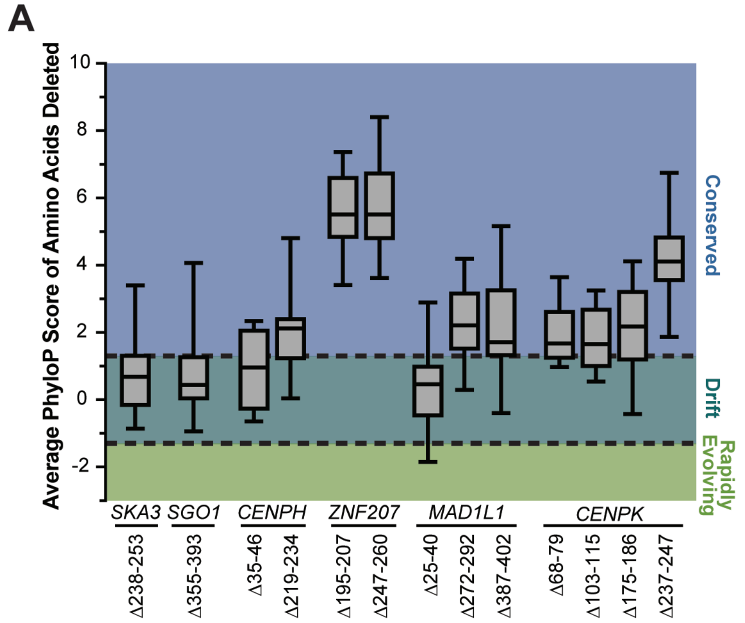

Figure S5. Functional validation and characterization of 11 high-resolution regions within 5 genes identified by tiling, continued. (A) Average PhyloP scores across 100 vertebrates for each codon within deletion mutants from figures 4 and S5 are plotted. Dashed lines denote sequences statistically (P<0.05) unchanged (PhyloP > 1.3) or rapidly changing (PhyloP < -1.3) sequences. Boxes represent median and quartiles while whiskers show range of datapoints. (B-C) Tiling profile, validation of proliferation phenotype, and assay of protein stability for (B) CENPH/Cenp-H and (C) SGO1/Sgo1. Tiling profiles are the same as Fig. 3 while also showing regions that were deleted. Cell proliferation was assayed as the cell number after knockout of endogenous protein in the presence (green) or absence (blue) of doxycycline in each cell line. The overlap of deletion and analysis methods shown as P = ProTiler, S = CRISPR-SURF, and T = TiVex. Cell numbers were normalized to the same cell line electroporated with a non-targeting control. Each dot is a biological replicate with bars showing median values and 95% confidence intervals. Paired tests were used to determine P-values. Steady state protein levels of wild type and mutant proteins was assayed by immunoblot in the presence of endogenous protein, using GAPDH as a loading control and exposed for a shorter interval.

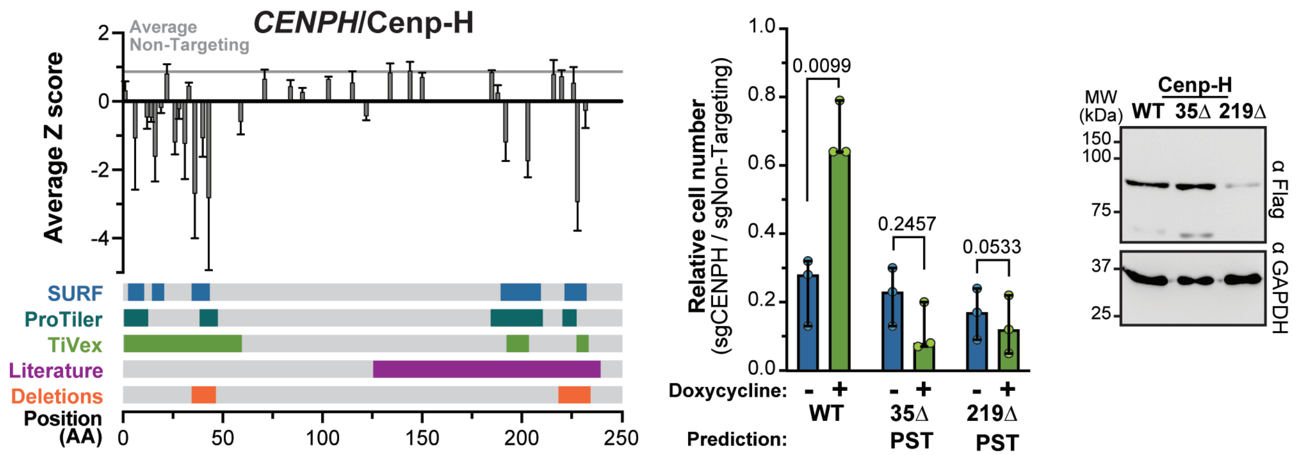

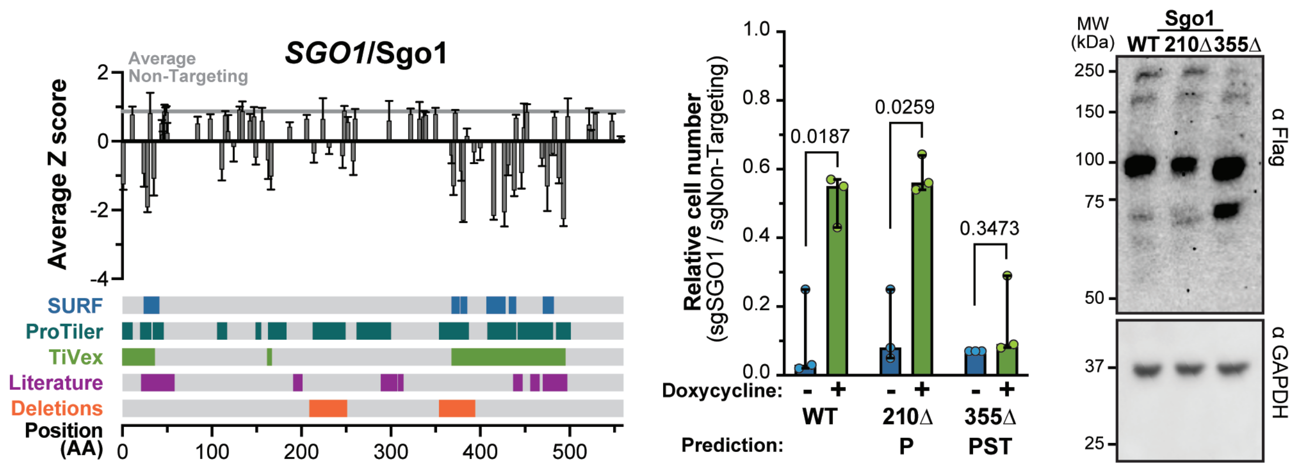

Supplementary Figure 6

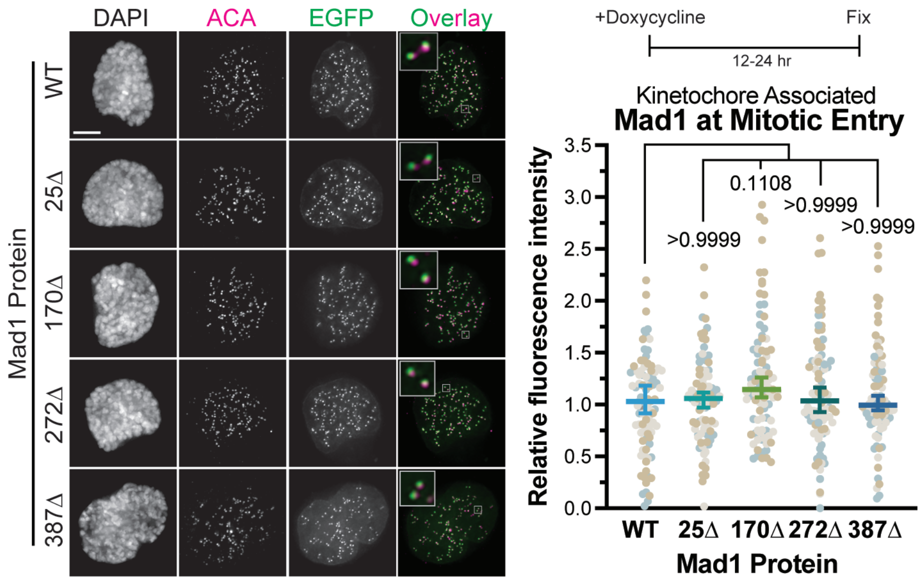

Figure S6. Mutations in Mad1 do not affect localization to kinetochores immediately following nuclear envelope breakdown. Kinetochore association of EGFP-Mad1 wild type and mutant fusion proteins was determined by the EGFP proximal to anti-centromere antibody (ACA) in the presence of endogenous Mad1 immediately after nuclear envelope breakdown. Representative images on left with quantifications on right. Each dot represents the average kinetochore signal from a single cell, cells from three biological replicates are colored differently. Dunn's multiple comparisons test was used to determine P-values. Scale bars are 1 μm, all averages and error bars in figure are median values and 95% confidence intervals.

Supplementary Figure 7

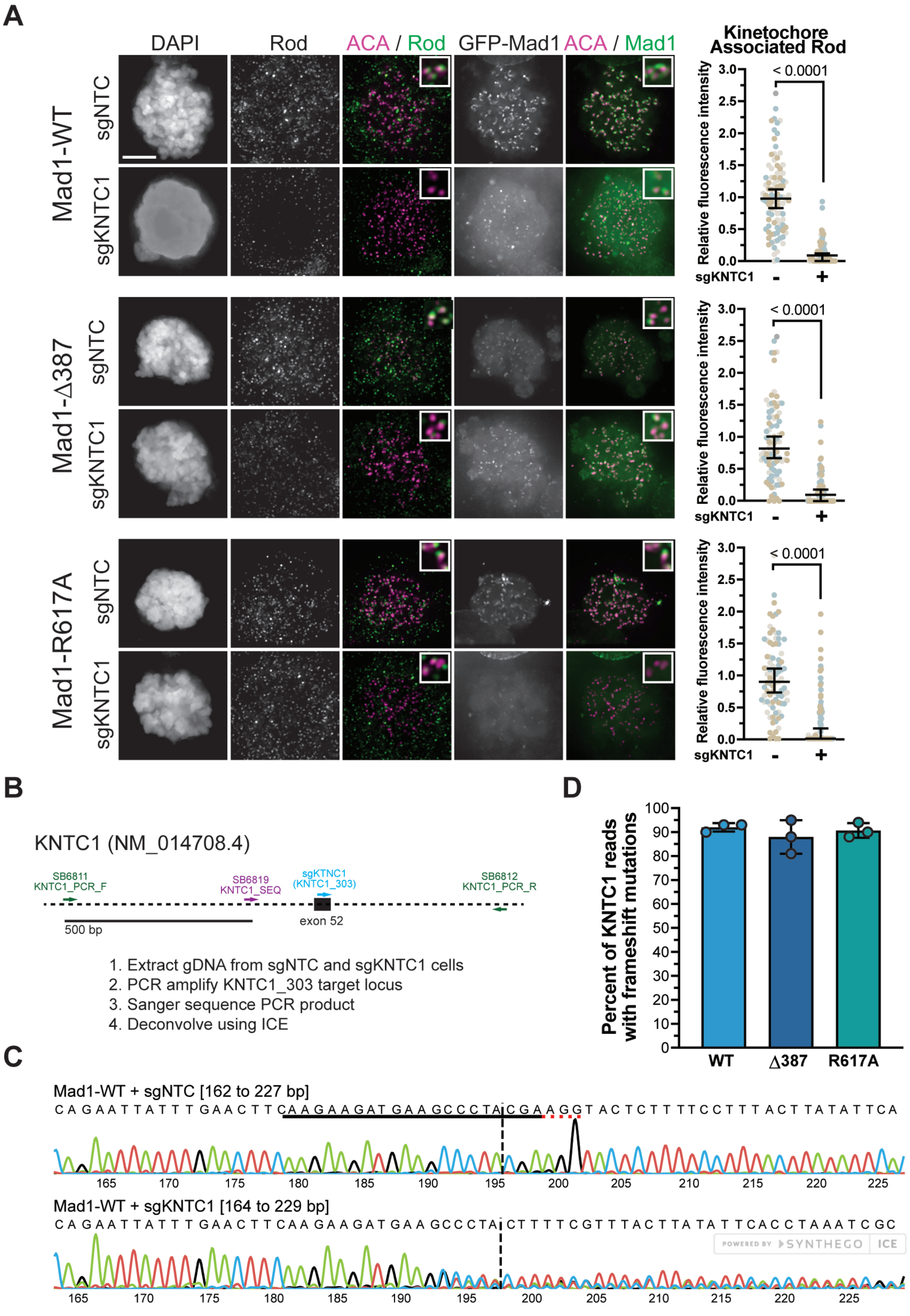

Figure S7. Electroporation of Cas9:sgKNTC1 RNPs causes robust deletion of KNTC1. (A) Kinetochore association Rod protein after KNTC1 knock out in cells from Figure 6 was determined by immunostaining for Rod and anti-centromere antibody (ACA). Representative images on left with quantifications on right. Each dot represents the average kinetochore signal from a single cell, cells from three biological replicates are colored differently. Dunn's multiple comparisons test was used to determine P-values. Scale bars are 1 µm, all averages and error bars in figure are median values and 95% confidence intervals. (B) Depiction of genomic site targeted by sgKTNC1 and primers used to validate deletion. (C) Sanger sequencing traces of PCR product from (B) in cells treated with sgNonTargetingControl (top) or sgKTNC1 (bottom). Edited sequences are aligned to the control population using sequence upstream of sgRNA target site (underlined sequence), which allows deconvolution of edited traces. (D) Deconvolution sequencing (B,C) was used to analyze cells treated with sgKNTC1 seven days after electroporation and percent of reads with frameshifting mutations is reported. Bars represent a mean value and error bars are 95% confidence intervals.

Supplementary Figure 8

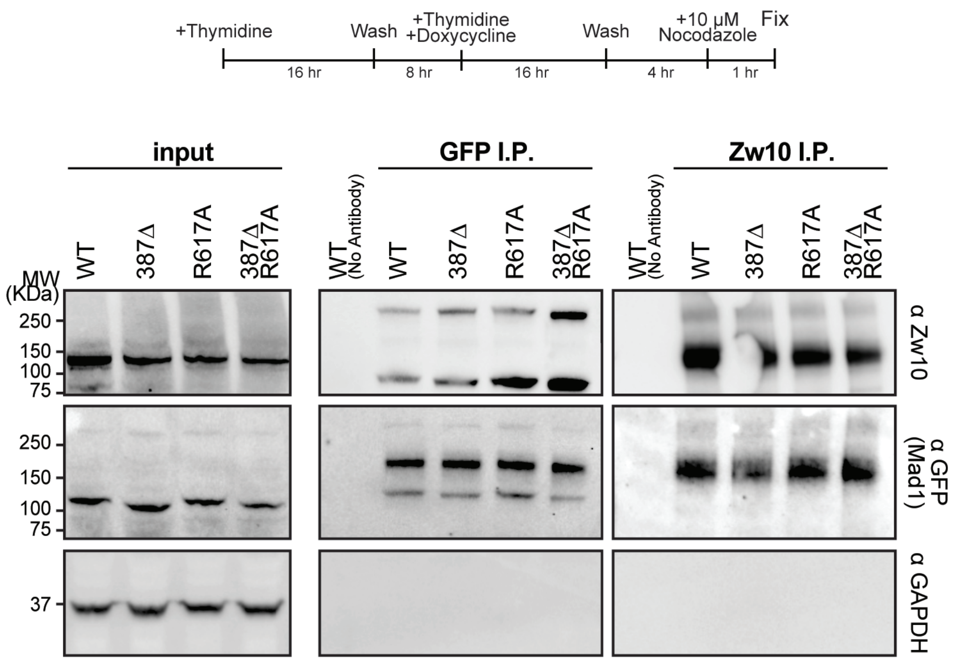

Figure S8. Mad1 mutants do not affect soluble interaction with Zw10. Immunoblots showing co-immunopurification of Mad1 proteins and RZZ complex member, Zw10. The same cell lysates (left) were used to purify exogenous EGFP-Mad1 (middle) or endogenous Zw10 (right) and then probe for GFP, Zw10, and GAPDH.
